# Application of real-time global media monitoring and ‘derived questions’ for enhancing communication by regulatory bodies: the case of human papillomavirus vaccines

**DOI:** 10.1186/s12916-017-0850-4

**Published:** 2017-05-02

**Authors:** Priya Bahri, Julianna Fogd, Daniel Morales, Xavier Kurz, on behalf of the ADVANCE consortium

**Affiliations:** 1 European Medicines Agency, 30 Churchill Place, Canary Wharf, London, E14 5EU UK; 2 Pharmacovigilance and Epidemiology Department, European Medicines Agency, London, UK; 3 Division of Population Health Sciences, University of Dundee, Dundee, UK

**Keywords:** HPV vaccines, Vaccines, Medicines, Media monitoring, Communication, Regulatory bodies, EMA

## Abstract

**Background:**

The benefit-risk balance of vaccines is regularly debated by the public, but the utility of media monitoring for regulatory bodies is unclear. A media monitoring study was conducted at the European Medicines Agency (EMA) concerning human papillomavirus (HPV) vaccines during a European Union (EU) referral procedure assessing the potential causality of complex regional pain syndrome (CRPS) and postural orthostatic tachycardia syndrome (POTS) reported to the authorities as suspected adverse reactions.

**Methods:**

To evaluate the utility of media monitoring in real life, prospective real-time monitoring of worldwide online news was conducted from September to December 2015 with inductive content analysis, generating ‘derived questions’. The evaluation was performed through the validation of the predictive capacity of these questions against journalists’ queries, review of the EMA’s public statement and feedback from EU regulators.

**Results:**

A total of 4230 news items were identified, containing personal stories, scientific and policy/process-related topics. Explicit and implicit concerns were identified, including those raised due to lack of knowledge or anticipated once more information would be published. Fifty derived questions were generated and categorised into 12 themes. The evaluation demonstrated that providing the media monitoring findings to assessors and communicators resulted in (1) confirming that public concerns regarding CRPS and POTS would be covered by the assessment; (2) meeting specific information needs proactively in the public statement; (3) predicting all queries from journalists; and (4) altering the tone of the public statement with respectful acknowledgement of the health status of patients with CRSP or POTS.

**Conclusions:**

The study demonstrated the potential utility of media monitoring for regulatory bodies to support communication proactivity and preparedness, intended to support trusted safe and effective vaccine use. Derived questions seem to be a familiar and effective format for presenting media monitoring results in the scientific-regulatory environment. It is suggested that media monitoring could form part of regular surveillance for medicines of high public interest. Future work is recommended to develop efficient monitoring strategies for that purpose.

**Electronic supplementary material:**

The online version of this article (doi:10.1186/s12916-017-0850-4) contains supplementary material, which is available to authorized users.

## Background

The benefit-risk balance of vaccines is frequently debated in the public domain and in particular in the media. These debates are linked on the one hand to the high expectations people have towards vaccines as one of the most successful health interventions to date and on the other hand to the phenomenon of vaccine hesitancy [1, 2]. The media debates have some aspects in common to all vaccines, but may also be driven by sentiments specific to certain vaccines [Karafillakis E, Larson H. The benefit of the doubt: a systematic literature review of perceived risks of vaccines in European populations. submitted]. Media attention may increase in cases of information on a new vaccine, an epidemic of a virus for which there is not yet a vaccine or the occurrence of a new vaccine-related concern. Regulatory bodies may be put in the spotlight, as they are in charge of vaccines licensure, safety surveillance, continuous benefit-risk assessment and, if needed, risk minimisation or other regulatory action. They also need to inform the public about the outcome of their assessments and provide advice on safe and effective use of vaccines [3].

As the communication process involves not only messaging, but also incorporating information from our surroundings and listening to others, mechanisms for effective listening need to be established. From the perspective of a regulatory body, listening should ensure that concerns expressed in the public domain are addressed in risk assessments [4], so that information, based on evidence and plausibility as well as on honesty over uncertainty, can be provided to the public and addresses their concerns. Furthermore, listening is an opportunity to collect data contributing to the body of evidence or its interpretation for risk assessment, such as data on how medicinal products are used by people. Through applying listening mechanisms, data on information needed by users of medicines enabling informed choice and safe use of medicines can be gathered too. In relation to vaccines, listening is a fundamental element of a new communication model which envisions communication as integrating with vaccine safety assessment and trust-building strategies [5].

Listening mechanisms available to regulatory bodies include directly interacting with members of the public (e.g. through working groups, public hearings, information contact points), conducting or reviewing research (e.g. surveys) and media monitoring. For the purpose of optimising communication about vaccines, media monitoring was proposed as early as the mid-1990s [6]. Based on this proposal and other vaccine communication experiences and research, media monitoring has been encouraged in the European Union (EU) vaccine pharmacovigilance guidance addressed to marketing authorisation holders and regulatory bodies [3]. The most recent media coverage study in Europe for a specific vaccine, conducted in Italy in relation to a seasonal influenza vaccine, recommended that public institutions should engage in prospective media monitoring [7]. While well-established medicines agencies conduct general media monitoring daily, the utility of medicinal product-specific media monitoring for regulatory bodies is as yet unclear.

### The case of HPV vaccines

When in July 2015 a EU referral procedure was initiated for human papillomavirus (HPV) vaccines [8], the European Medicines Agency (EMA) decided, given frequent media debates about vaccines, to conduct and evaluate, for the first time, medicinal product-specific media monitoring to support preparations for communicating with the public about the assessment and outcome of the referral procedure. The assessment of HPV vaccines was considered a specifically important test case for media monitoring, as a public debate with a wide range of topics about these vaccines had already accompanied their licensure and launch in 2006. The debate then had been characterised by celebrating future reduction in mortality from HPV-related cervical cancer on the one hand, but also raised a number of concerns on the other, mainly about long-term effectiveness and benefit [9–14] as well as safety [9, 11, 13–18]. Beyond benefit and safety, social concerns had arisen at the time too, speculating that HPV vaccination would encourage increased or unsafe sexual activity of the young [13–15].

The media monitoring study was initiated in September 2015 during the EU referral procedure assessing a potential causal association between HPV vaccines and the suspected adverse reactions of complex regional pain syndrome (CRPS) and postural orthostatic tachycardia syndrome (POTS). Cases of CRPS and POTS symptoms occurring after HPV vaccination had been reported to the authorities [8]. The assessment was carried out by the Pharmacovigilance Risk Assessment Committee (PRAC), the EMA’s scientific committee responsible for monitoring and assessing safety issues for human medicines in the EU and providing recommendations for risk minimisation or other regulatory action as necessary. The PRAC is composed of members from each of the 28 EU member states, Iceland and Norway as well as additional scientific experts and healthcare professional and patient organisation representatives. Raising a safety issue with a medicine at EU level through a referral procedure results in a regulatory decision being implemented in all member states, Iceland, Norway and Liechtenstein. The PRAC recommendations on the referral for HPV vaccines were finalised and published in November 2015. The EMA has a communication department which prepares communication on all safety-related referral procedures and responds to queries from the public, including journalists.

## Methods

### Aim and objectives

The aim of this study was to evaluate whether prospective real-time media monitoring for specific medicinal products has the potential to enhance communication in terms of proactivity and preparedness for information provision to the public, in particular by a regulatory body.

Therefore, the following objectives were defined:

1. Develop a method for medicinal product-specific media monitoring in a real-life scenario.
2. Identify, through media monitoring, areas of concerns, information needs and expectations of the public in relation to the EU referral procedure assessment of HPV vaccines and take them into account when preparing communications.
3. Evaluate the utility of media monitoring to enhance communication.

It is *not* the objective of this article to provide information on the safety profile of HPV vaccines or to explain the outcome of the EU referral procedure on HPV vaccines and CRPS/POTS, as these are presented elsewhere [8, 19].

### Study period

The study period started on 7 September and lasted until 23 December 2015, i.e. two months before and after the PRAC meeting, where the HPV vaccines assessment was scheduled for discussion and finalisation of the PRAC outcome occurred.

### Search strategy

The media monitoring was conducted using Vuelio®, a media intelligence system which sources media content from a wide range of news and social media outlets worldwide in real time [20]. The following search settings were applied to all available online news stories and blog posts:

- *media types*: worldwide online health, science, news and tabloid media, online websites of television channels and blogs (no restriction was set by e.g. media type, size of readership or size of country of origin, as it was considered that some relevant news would possibly be disseminated only by media with a small reach or by blogs)
- *search terms*: the colloquial and technical terms as well as tradenames: “HPV vaccin*”; “papilloma W/3 vaccin*”; “cervical cancer vaccin*”; “HPV jab”; “papilloma jab”; “cervical cancer jab”; “Gardasil”; “Cervarix”; “Silgard” (the asterisk symbol * indicates that the ending of ‘vaccin’ could vary (e.g. vaccin*e*, vaccin*ation*); ‘W/3’ (or W/2, W/4, W/5, etc.) indicates the number of words that could be inserted between two word elements)
- *languages*: all official EU languages except for Irish and Maltese, i.e. 22 languages (the search terms were translated by native speakers at the EMA).

### Daily media screening

Due to the application of a broad search strategy, the Vuelio system identified a large number of news stories and blog posts. These articles were first screened daily by JF for topic relevance, applying the following exclusion criteria, namely if the article:

1. Is about business or financial news only; or
2. Contains no information about HPV vaccines (but was identified by the system due to the different meanings of the multilingual search terms in other languages, in particular of the abbreviation “HPV”).

Articles in languages other than English were initially screened using Google Translate®, and for those considered critical, summary translations were provided by native speakers at the EMA.

### Weekly media content analysis

Weekly charts of the global media screening output by date and by country were prepared by means of the Vuelio system, and weekly key topic summaries containing references to articles were created by JF, applying the following inclusion criteria, namely if the article:

1. Covers a topic about HPV vaccines with higher weekly media coverage than other topics;
2. Discusses an adverse event following immunisation, including personal experiences of individuals or the risk-benefit balance of HPV vaccines;
3. Reports about studies on HPV vaccines;
4. Reports about advocacy or parents’ groups active in relation to HPV vaccines; or
5. Mentions the EMA, the PRAC or an assessment, referring to the EU referral procedure for HPV vaccines.

Based on the key topic summaries, a content analysis of the included articles was undertaken by PB, resulting in formulation of considerations for communication preparations. Content analysis is a qualitative study design, and as such appropriate for exploring phenomena not yet well understood. It is an interpretative approach concerned with understanding the meanings that persons attach to actions, decisions, beliefs and values within their social world, as well as the mental processes persons use to make sense of the world around them [21]. The considerations for the communication preparations reflected upon concerns, information needs and expectations of the public in relation to HPV vaccines and their benefit-risk assessment by regulatory bodies as well as experiences and advice blogged by healthcare providers about the type of questions they are frequently asked by parents and how they respond. This content analysis therefore consisted of interpreting the explicit content as well as connotations and underlying assumptions implicit to the content and identifying questions raised explicitly or implicitly, including those raised due to lack of knowledge or anticipated to be raised once more information would be provided in the public domain. Lack of knowledge in this context refers to knowledge of scientific nature or about regulatory processes beyond the scope of common knowledge.

The charts, the key topic summaries and the considerations were presented in weekly media monitoring reports.

### Presentation and categorisation of media content as derived questions

A cumulative review of the weekly media content analyses was performed by PB in the month prior to the scheduled finalisation of the PRAC assessment for supporting the preparations of the communication for the EU referral outcome. Identified concerns, information needs and expectations of the public were used by PB to derive questions from the media content. These were worded in an abstract manner, i.e. often differently from the questions raised explicitly in the media or were worded anew if the concern had not been presented in the media as a question at all, but its discussion implied a question or lack of knowledge which was considered as important for regulatory bodies to be prepared to answer or even fill with information proactively. The derived questions were written using language with terms familiar to colleagues in the regulatory and scientific domain involved in risk assessment or communication about the outcome of the assessment. PB grouped the derived questions into theme-based categories which had emerged from reviewing the content and organising these text data. Each category was allocated a high-level derived question and further sub-questions on specific aspects.

This approach to content analysis corresponds to an inductive method, i.e. a method by which concepts for categorising the content are not defined in advance or derived from other research but created through the content analysis itself, and where abstraction is applied through generating categories. This has been described as a conceptual and empirical challenge. An inductive approach is recommended where prior knowledge on the content or aspects relevant to the purpose of the content analysis is limited. Instead of relying on pre-defined categories, this flexible and sensitive approach allows one to go beyond a simplistic content description towards a deeper and new understanding of complex phenomena [22], here the meaning of what has been expressed explicitly and implicitly in the media. The inductive approach also allowed for creation of categories specifically relevant to a regulatory body.

### Evaluation of utility

For evaluating the utility of medicinal product-specific media monitoring for regulatory bodies, the following was undertaken: (1) obtaining feedback in person from colleagues within the EU regulatory network using the media monitoring results; (2) reviewing the summary of PRAC recommendations (PRAC outcome) with a view on whether and how the derived questions were addressed; and (3) validating the predictive capacity of the derived questions by comparing the derived questions retrospectively with the queries raised by journalists towards the EMA during the study period.

The authors were not involved in the activities of the PRAC or the EMA communication department during the time of the referral procedure, had no influence on the assessment or the drafting of the outcome statement (other than through providing the results from the media monitoring) and had no knowledge of the journalist queries raised during the study period. The authors can therefore be considered ‘blinded’ for the purpose of the study.

## Results

### Media monitoring

**Fig. 1:**
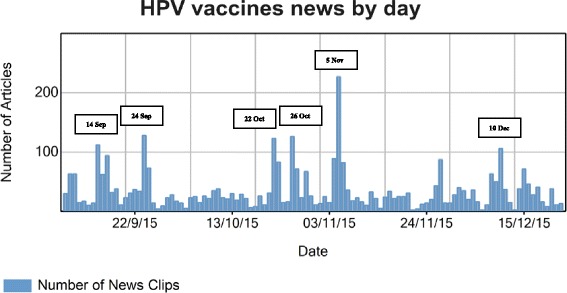
Time chart depicting volume of worldwide media coverage from 7 September to 23 December 2015 by day and identifying peaks (generated by the Vuelio® system)

A total of 4230 articles (‘news clips’, i.e. news stories (3737) and blog posts (493)), were collected worldwide during the monitoring period (after applying the exclusion criteria). These originated from 2124 media outlets (see Additional file 1). The highest media coverage for Europe in terms of absolute numbers of articles was found in Denmark (626, based on the country allocation in the Vuelio system), whose authorities received reports for the suspected adverse reactions of concern at higher rates and initiated the EU referral procedure [8]. An analysis of intensity of the worldwide coverage for HPV vaccines by day identified six peaks with more than 100 articles in a day. The highest peak occurred on 5 November 2015 when the PRAC recommendations on the EU referral procedure were published by the EMA (Fig. 1). JF related these peaks to the triggering events identified through the media screening (Table 1) and compiled all key topics considered major over time in terms of the volume of media coverage or the relevance of the topic in relation to HPV vaccine risks, benefits, marketing authorisation or immunisation policy decisions (Table 2).

**Table 1:** Triggering events of peaks of the worldwide media coverage on HPV vaccines from 7 September to 23 December 2015^a^

Peak time	Peak-triggering event
1^st^ peak, 14 September 2015	Study published by the Agence Nationale de Sécurité du Médicament et des Produits de Santé (ANSM, the French medicines agency) and the French health insurance, concluding that HPV vaccines do not increase the risk of autoimmune disorders but suggesting increase of the risk of Guillain-Barré syndrome [30]
	Call by two Republican Party lawmakers in the USA towards schools to oppose mandatory HPV vaccination of middle school students in Rhode Island [31]
2^nd^ peak, 24 September 2015	Statement of the Catholic bishop in British Columbia, Canada, saying abstinence is the only healthy choice over HPV vaccination [32]
	Announcement in Denmark of the replacement of Gardasil® by Cervarix® in the national HPV immunisation programme [33]
	Report claiming that 1500 girls in Denmark have suspected adverse reactions to HPV vaccines [34]
3^rd^ peak, 22 October 2015	Study published in the journal *Epidemiology, Biomarkers and Prevention* concluding that a quarter of doctors in the USA do not strongly endorse HPV vaccination [35]
4^th^ peak, 26 October 2015	Statement of the International Papillomavirus Society (IPVS) endorsing the use of HPV vaccines [36]
	Concerns in Denmark on the marketing authorisation holder’s restrictive search strategy on adverse effects of HPV vaccines [37]
	Study published by the US Centers for Disease Control and Prevention (CDC) about low HPV vaccine uptake among adolescent males in the USA [38]
5^th^ peak, 5 November 2015	Publication by the EMA of the PRAC outcome of the referral procedure, concluding that the evidence does not support a causal association between HPV vaccines and CRPS or POTS [23]
6^th^ peak, 10 December 2015	Statement by Health Canada referring to a review of international research data suggesting that there are no new risks associated with Gardasil® and that it can be used safely [39]

^a^The references do not provide all news stories or blog posts; they provide the key source as far as identifiable or selected examples of articles

**Table 2:** Major topics discussed in the media for HPV vaccines worldwide from 7 September to 23 December 2015^a^

Topics
Experiences of female adolescents with suspected adverse reactions of HPV vaccines and beliefs in causal association with HPV vaccines [40]
Number of suspected adverse reaction reports received by the Danish authorities [34] and concerns in Denmark on the marketing authorisation holder’s restrictive search strategy on adverse effects of HPV vaccines [37]
Statements from parents claiming that they were not sufficiently informed about the adverse reaction profile of HPV vaccines before their decision-making on vaccination [41–43]
Questions about safety and benefits of HPV vaccines [44, 45]
Study on misleading information on HPV vaccines on the Internet [46]
Lack of treatment options for CRPS and POTS [47]
Activities of anti-HPV vaccination groups and opinion leaders [32, 48–53]
Protest by parents and activities by politicians against mandatory HPV vaccination in Rhode Island, USA [31, 54]
Call by the Irish government for investigations on suspected adverse reactions with HPV vaccines [42]
Continued suspension of HPV vaccination recommendation by the Ministry of Health in Japan [55]
USA presidential candidate Donald Trump claiming a causal association between vaccines and autism [56]
Replacement of Gardasil® by Cervarix® in the national HPV immunisation programme in Denmark [33]
Support to HPV immunisation programmes [36, 57–59]
Reassuring safety and/or benefit data supporting HPV vaccination policies [19, 30, 36, 39, 60]
Protection against genital warts by HPV vaccination [61, 62]
Protection against mouth cancer by HPV vaccination and the importance of immunisation of boys [63]
Low HPV vaccine uptake by female and male adolescents in the USA [38, 64]
Responsibility of physicians for low HPV vaccination rates [35]
Discussion about appropriate HPV vaccination age [65]
Mainly neutral, but also some negative media coverage of the PRAC recommendation on the EU referral procedure on HPV vaccines [3], in particular in Denmark and Sweden [66, 67]
Need for further independent studies on the association between HPV vaccines and CRPS/POTS [68–70]

^a^ The references do not provide all news stories or blog posts; they provide the key source as far as identifiable or selected examples of articles

The media content analysis (after applying the inclusion criteria) by PB identified patterns over time: While many debates remained nationally contained, some topics ‘travelled’, in particular between Scandinavian countries and those countries with active parents’ groups, such as Demark, Ireland and the UK. There was also a change in focus; in particular in Denmark the debate moved from presenting personal stories to additionally including scientific and policy-related points. There were increasingly references to scientific publications on safety aspects, and overall the debate turned from questioning vaccine safety as such to doubting the trustworthiness of the data, the pharmaceutical industry as a data source and the integrity of the regulatory and other public health bodies in collecting and assessing data. Some debates claimed that the EMA and its scientific committees do not exercise separation between pre- and post-authorisation decision-making and rely on data provided by industry rather than requiring data from independent sources. This led to including in the respective weekly considerations for communication preparations that regulatory bodies should be prepared to answer in detail how they ensure the legally demanded independence of their work and manage potential conflicts of interests and how the pharmaceutical industry is inspected for their compliance with legal requirements.

Based on the cumulative review of the concerns, information needs and expectations of the public identified through the weekly media content analyses, 50 derived questions were formulated, which could be categorised into 12 themes with a high-level question each (12 questions) and sub-questions on specific aspects (38 questions) (Table 3).

**Table 3:** Derived questions based on a content analysis of the media coverage for HPV vaccines worldwide from 7 September to 22 October 2015 and categorised by themes

Categories: themes and high-level questions	Sub-categories: additional aspect-specific questions
Theme 1 - Assessment scope: 1.0. What is the scope of the assessment conducted for the EU referral procedure for HPV vaccines?	1.1. Why does the procedure focus on CRPS and POTS as defined by complex and difficult-to-apply/ascertain case definitions?
	1.2. Why have concerns over autoimmune diseases with HPV vaccines been excluded from the assessment?
	1.3. Why does the evaluation not cover the entire benefit-risk balance of HPV vaccines?
Theme 2 - CRPS and POTS case data: 2.0. What kind of case reports of CRPS and POTS in association with HPV vaccines have been reviewed by the authorities, and how?	2.1. How many case reports of CRPS and POTS in association with HPV vaccines have been received by the authorities, who reported the cases to the authorities and who are the primary reporters?
	2.2. Who confirmed the cases as CRPS and POTS cases?
	2.3. How many cases have been received with symptoms of, or similar to those of, CRPS and POTS but have not met the criteria of the case definitions, how were these cases reviewed/followed up and how did they have an impact on the assessment outcome?
	2.4. Have all reported cases been followed up by the authorities in order to obtain more information (to allow for causality assessment)?
	2.5. What is the outcome of the analysis of data recorded in EudraVigilance (the adverse reaction database of the EU regulatory network) requested by parents who have participated in the EMA meeting with concerned vaccinees and parents to present their concerns and experiences?
	2.6. How were the cases reviewed that had been submitted to the authorities by the parents’ groups as invited by the EMA?
Theme 3 - Frequency assessment: 3.0. What are the reporting rates and actual frequencies of CRPS and POTS in association with HPV vaccines?	3.1. How are these frequencies calculated?
	3.2. Where have background frequency data been obtained from, and how confident can one be in their accuracy?
	3.3. What is the likely magnitude of underreporting, and has a sensitivity analysis been performed for the observed/expected analysis to take underreporting into account?
	3.4. Why are the reporting rates for (any) adverse reactions higher for HPV vaccinesthan for other vaccines?
Theme 4 - Other (i.e. not case) CRPS and POTS data: 4.0. What kind of data has been reviewed for the EU referral procedure for HPV vaccines in addition to individual case reports?	4.1. What is the nature of these data, and who provided them?
Theme 5 - Assessment of causal association: 5.0. How has the assessment of CRPS and POTS in possible causal association with HPV vaccines been performed?	5.1. Have all potential aetiological pathways been investigated, e.g. autoimmune pathway and impact of female hormones on susceptibility for autoimmune disease?
	5.2. How has causal association been ruled out?
Theme 6 - Overall safety and other safety concerns: 6.0. What are the overall safety database and safety study results for HPV vaccines?	6.1. What was the knowledge base at the time of granting the marketing authorisation, and were the vaccines sufficiently tested at the time?
	6.2. How are data assessed for autoimmune diseases, including multiple sclerosis and Guillain-Barré syndrome?
	6.3. How are data assessed for infertility, miscarriage and stillbirth?
Theme 7 - Aluminium: 7.0. What is the knowledge about the safety of aluminium/AS04 as adjuvant?	7.1. What are the plasma levels for aluminium after vaccination with current HPV vaccines and with the future Gardasil-9® compared to typical food intake?
	7.2. How does the clearance process of aluminium in the human body work?
	7.3. Since when has the rate of autism diagnosis been increasing, and is there a temporal association with the use of aluminium in vaccines?
	7.4. What is known about a link between AS04 (aluminium hydroxide + monophosphoryl lipid A) and autism?
	7.5. How similar is AS04 to AS03 (squalene + dl-α-tocopherol + polysorbate 80), which is the adjuvant in Pandemrix® for which cases of narcolepsy were reported as suspected adverse reactions?
Theme 8 - Data trustworthiness: 8.0. Are the data for the EU referral procedure for HPV vaccines trustworthy?	8.1. What safeguards are there to ensure that marketing authorisation holders do not manipulate data they submit to the authorities?
	8.2. Have data been solicited by the authorities from independent sources?
Theme 9 - Assessment standards and integrity: 9.0. How can it be demonstrated that signal detection, risk evaluation and decision-making have been performed to highest standards during the EU referral procedure for HPV vaccines?	9.1. Have the authorities taken seriously the vaccinated females experiencing CRPS and POTS?
	9.2. How do the authorities manage their conflict of interests?
	9.3. Why was the signal of CRPS and POTS with HPV vaccines not identified earlier, and why was the referral procedure only initiated at the request of Denmark and not earlier by the EMA?
	9.4. Why did the EMA not apply the precautionary principle and suspend the vaccine while investigations were ongoing?
Theme 10 - Benefit: 10.0. What is the knowledge on the benefit and effectiveness of HPV vaccines?	10.1. How does the vaccine intervene protectively in the pathway of cancer development?
	10.2. How long is the vaccination effective in vaccinees, and what should vaccinees do after immunity has decreased?
	10.3. What is the potential of strain replacement, and how will this have an impact on cancer rates?
Theme 11 - Benefit-risk balance: 11.0. What does the statement ‘the benefits outweigh the risks’ mean?	11.1. Is this statement only applicable at population level or also at the individual level, and does a positive benefit-risk balance apply to all potential vaccinees or are there individuals to whom the statement does not apply?
	11.2. How are healthcare professionals provided with information so that they can communicate well with potential vaccinees and parents about the individual benefit-risk balance?
Theme 12 - Further steps and research: 12.0. What will the impact of the EU referral outcome be, and will further research be done?	12.1. How do vaccine evaluations by the authorities have an impact on immunisation policies?
	12.2. What kind of further research will be done, and what will be the study objectives?
	12.3. How will independence of this research be ensured?

In addition to the theme categorisation and identifying patterns of flow and focus over time, the media content analysis allowed for an understanding of some of the motivations and expectations of parents. The parents who expressed their opinions in the media considered the information they had received on vaccine risks in general as insufficient. Parents who suspected that their daughters had been harmed by HPV vaccination mainly wanted to provide case information to the authorities, obtain *support* and treatment within the governmental health insurance as well as remedy *the lack of respect* their daughters feel that they experience in relation to their condition. Some also requested ending the HPV vaccination programme or wanted other parents to be provided with information about the ongoing EU referral review prior to giving an informed consent to vaccination. Giving special attention to respectfully acknowledge the health status of the patients, regardless of what the outcome of the EU referral would be, was therefore added to the considerations for preparing communication.

### Utility

The users of the media monitoring results were the EMA communication department, the PRAC and the EU member states. Through the media monitoring, the media office of the EMA communication department became immediately aware of emerging issues which needed attention. The weekly media monitoring reports and the derived questions were circulated within the EMA and to the PRAC.

*(1) Feedback from users of the media monitoring results*

PRAC members from those EU member states leading the EU referral assessment and/or having higher national media activity or those PRAC members contributing special expertise in vaccines or communication (i.e. five PRAC members) were asked in person for feedback. They noted that the weekly media monitoring reports enhanced their communication preparedness for possible questions from the public they had not envisaged before. Members from the Scandinavian countries remarked that the reports helped them to put the media attention at national level in a broader European and global context. When taking a specific look at the weekly considerations in early October 2015, the PRAC members leading the assessment confirmed that all identified concerns and information gaps relating to CRPS and POTS as voiced by the public would be covered by the ongoing assessment. It was agreed that the broader public concerns, such as those about aluminium-containing adjuvants, had been evaluated in the context of previous assessments. This provided useful reassurance that no additional data reviews would be necessary to respond to anticipated questions from the public.

The medical writers of the EMA communication department stated that they were guided by the derived questions as to which information items from the assessment to include proactively in the summary of PRAC recommendations [23], i.e. the public statement on the PRAC outcome for website publication and dissemination to the EU regulatory network, its international partners, relevant patient and healthcare professional organisations and journalists (see *(2)* below). The derived questions guided these colleagues further as to which information to include in the talking points, prepared for the EMA itself as well as for the authorities in EU member states, to enable prompt provision of accurate and consistent information in response to external requests, including those from journalists (see *(3)* below). The talking points were also used by senior EMA colleagues to prepare for attending, upon invitation, a discussion on HPV vaccines at the Danish parliament in December 2015. The identification of the pattern of the public debate becoming increasingly focussed on scientific and policy-related points, particularly in Denmark, was considered to be especially helpful for these preparations.

*(2) Review of the impact of the derived questions on the summary of PRAC recommendations*

The summary of PRAC recommendations [23] showed that, in addition to reporting upon the assessment outcome in general, specific information had been included addressing all categories of derived questions relating to the assessment of the suspected CRPS/POTS reported for HPV vaccines, i.e. categories 1 to 5. The medical writers had selected 7 questions out of 21 (33%), and addressed them as follows:

- The PRAC *reviewed the published research, data from clinical trials and reports of suspected side effects from patients and healthcare professionals, as well as data supplied by Member States*; (addresses derived questions 2.0. and 4.1.).
- The PRAC *took into account detailed information received from a number of patient groups that also highlighted the impact these syndromes can have on patients and families* (addresses derived question 2.6.).
- *Symptoms of CRPS and POTS may overlap with other conditions, making diagnosis difficult in both the general population and vaccinated individuals.* and *The PRAC noted that some symptoms of CRPS and POTS may overlap with chronic fatigue syndrome (CFS, also known as myalgic encephalomyelitis or ME). Many of the reports considered in the review have features of CFS and some patients had diagnosis of both POTS and CFS. Results of a large published study that showed no link between HPV vaccine and CFS were therefore particularly relevant.* (addresses derived questions 1.1. and 2.3.).
- *…available estimates suggest that in the general population around 150 girls and young women per million aged 10 to 19 years may develop CRPS each year, and at least 150 girls and young women per million may develop POTS each year. The review found no evidence that the overall rates of these syndromes in vaccinated girls were different from expected rates in these age groups, even taking into account possible underreporting.* (addresses derived questions 5.0. and 3.3.).

The quotes show that detailed medical and methodological aspects such as those regarding patient impact, case ascertainment and adverse event underreporting, that are usually not included in summaries of PRAC recommendations, were included in the summary on HPV vaccines (however it is routine for the EMA to publish these aspects in the full assessment reports).

With regard to the tone of the summary of PRAC recommendations, the evaluation identified words intended to express commitment and diligence towards patients with CRPS and POTS and to acknowledge the seriousness of what they were experiencing. The summary highlighted that the scientific review was *detailed*, performed *thoroughly* and in consultation with *leading experts*. It further stressed that CRPS and POTS *can severely affect the quality of life*. This kind of wording is not the routine way of expression of a regulatory body, and a comparison confirmed that other summaries published by the EMA in 2015 were devoid of empathy.

*(3) Capacity of derived questions to predict queries from journalists*

The retrospective comparison of the queries from journalists to the EMA with the derived questions validated the predictive capacity and utility of media monitoring for communication preparations.

At the EMA press briefing about the PRAC outcome on 5 November 2015, four queries were raised by journalists [24], which were all predicted by the derived questions and therefore addressed in the talking points. This enabled EMA colleagues to provide well-informed responses promptly at the briefing. The queries related to themes 2, 3, 4, 5 and 12.

Before and after publication of the PRAC outcome, the EMA was contacted by journalists, as it is often the case for EU referral procedures on safety issues. While the referral procedure for HPV vaccines was ongoing, journalists often requested the timetable for finalising the assessment or asked for access to documents or interviews. A total of 16 journalist queries received during the entire study period contained actual questions, 9 before and 7 after the publication of the PRAC recommendations on 5 November 2015. These queries all corresponded with the derived questions (queries before 5 November: 1.0., 1.1., 2.0., 2.3., 3.0., 4.0, 5.0., 9.0, 9.2., 9.4., 10.0., 10.2. and 10.3., and after 5 November: 1.1., 2.0., 2.3., 3.0., 3.2., 3.3., 4.0., 7.0., 9.2., 10.0. and 12.0.). However, in some instances, the level of detail of the queries from journalists was not predicted. Clarifications on how referral procedures work in general were also frequently requested before and after publication of the PRAC outcome. The EMA responded to all queries from journalists, whether or not they were included in the talking points. The EMA’s declaration of interest policy and actual experts’ declarations had already been made accessible by the public [25]. For the comparison of the queries from journalists with the derived questions for validation purposes, the time period after the publication of the PRAC recommendations on 5 November was the relevant one, as the media monitoring results were used for preparing communication on the PRAC outcome, i.e. to inform the drafting of the statement on the PRAC recommendations and the talking points.

## Discussion

The article reports on the first time experience of the EMA with medicinal product-specific media monitoring and in-depth content analysis. The aim of this study was to evaluate whether such prospective real-time media monitoring has the potential to enhance communication in terms of proactivity and preparedness for information provision to the public, and the study results contribute to filling the current knowledge gap as to whether regulatory bodies, and other stakeholders in medicines, should engage in medicinal product-specific media monitoring. To the authors’ knowledge, no other study has yet been conducted within a regulatory body and evaluated the utility of product-specific media monitoring. Published studies on vaccines in the media often review media coverage retrospectively. Here, media monitoring has been conducted prospectively in real time.

As this study investigated media monitoring for the purpose of supporting communication proactivity and preparedness, it was not the objective to quantify media coverage or obtain a representative picture of the public debate. Instead, it was the objective to gain an understanding of concerns, information needs and expectations of the public, in the sense of all groups of the public, since a regulatory body serves all citizens and needs to understand which kind of information on scientific evidence and regulatory processes is missing in the public domain.

Through conducting the study, a method for media monitoring has been developed, including a new approach to analysing and presenting media coverage by translating the media monitoring findings into derived questions for regulators to consider. With this approach, concerns, information needs and expectations towards regulatory bodies that have been discussed in the public domain explicitly, implied, raised due to a lack of knowledge, or anticipated to be raised once more information has been published, have been expressed in the form of specific questions in scientific language and with reference to regulatory policies and procedures. Question formats are familiar to those in the scientific-regulatory domain and seemed to be an effective way to provide feedback from the public domain to those in charge of assessing medicines or defining messages on assessment outcomes. While media monitoring seeks to understand relevant mental models (i.e. explanations of someone's thought process about how something works in the real world [26]) prevalent in the public domain, the translation of the media monitoring findings into derived questions intends to make the findings fit into the thought processes of the scientific-regulatory domain, whilst helping to focus communication on specific pieces of information either missing, or expected, in the public domain. Some vaccine safety experts have raised questions as to whether listening and providing feedback in response to unsubstantiated concerns voiced by the public could risk what has been referred to in the literature as the ‘social amplification of risk’ [27]. While recognising this risk, genuinely listening with openness to concerns voiced by the public and responding honestly and with transparency is considered essential for building and sustaining trust [28], a fundamental principle in relation to matters of public good.

The study used a large data set of worldwide news stories and blog posts. Although the study was conducted for only one regulatory procedure, it was one of major importance. However, the study has some limitations in the utility evaluation. Despite media monitoring findings being widely disseminated via the PRAC to all EU member states and to the EMA communication department, feedback was only obtained from those most closely working on the EU referral procedure itself. However, more systematic evaluation methods included reviewing the impact of the media monitoring findings on the public PRAC outcome statement, and comparing the derived questions with the queries from journalists, which revealed their predictive capacity of 100% in terms of themes. The study did not include a survey or obtain views directly from young people, parents or healthcare professionals in the EU. It is assumed that if members of the public search for information from authorities, they usually view the websites of the authorities in their own country rather than the EMA website, given that the EMA website is mainly visited by professionals in pharmaceutical industry, regulatory authorities and the media. Further evaluation could include studying how the outcome of the EU referral procedure on HPV vaccines was reported in the news and social media, whether public concerns persisted and were discussed in the light of the assessment by PRAC, or whether journalists felt satisfied by the information published by the EMA in terms of scope, format and language. Correlations between information from regulatory bodies, media coverage and vaccination rates among the population would be interesting for establishing the impact of communication by regulatory bodies on medicines use behaviours.

In terms of resources, 165 working hours of a communication specialist (JF) and 75 working hours of a medicines safety specialist (PB) were used for the media screening and content analysis in 2015. This equals 0.34 and 0.15, respectively, full-time equivalents per month. In order to use resources for media monitoring efficiently, the experience from this study suggests that limiting the number of languages monitored to English and to languages from those countries with high relevant media coverage could be sufficient, but further work is required to clarify the most efficient process to support routine implementation. The use of exclusion terms (e.g. “budget”, “profit”) to automatically, rather than manually, exclude articles (e.g. financial news) had been discussed when setting up the search strategy, but was not implemented because of concerns around excluding articles about important policy and trust issues. The study showed that concerns about profit-driven bias and expectations for independent data gathering and assessment were indeed voiced by the public and important to be addressed by regulatory bodies through transparency of their procedures. In order to investigate if resource efficiency can be augmented by automated media screening, further work focussing on developing hierarchical or conditional search algorithms with increased specificity and without losing sensitivity is recommended. Now that a potentially workable method for media content analysis has been developed, future media monitoring for other medicinal products could make efficient use of the available method.

The study results contribute to the Accelerated Development of VAccine benefit-risk Collaboration in Europe (ADVANCE), a private-public consortium aiming to establish a reliable, valid and tested framework for providing rapidly robust data and scientific evidence on vaccine benefits and risks in Europe. As part of this framework, ADVANCE is developing recommendations for communication strategies in relation to vaccine benefit-risk monitoring [29].

The following principles and actions on communication are therefore recommended in general for any stakeholder assessing vaccines (e.g. regulatory and public health authorities, industry or academia) and for ADVANCE in particular:

- Efficient media monitoring should be built into the process of vaccine benefit-risk monitoring, and benefit-risk assessment should ensure the provision of responses to all safety concerns, including those debated in the public domain.
- Explanations on methods for benefit-risk monitoring and assessment should be provided in a language understandable to the public, and should be developed and ideally be tested with a view to explaining how the method works and how robust the results are.
- Given that conflicts of interests are a major concern voiced by the public, procedures to ensure unbiased monitoring and assessment as well as, if applicable, the mechanisms of public-private partnership (PPP) governance models (as envisaged by ADVANCE as one option for a future platform for vaccine benefit-risk monitoring) need to be proactively communicated to the public.

## Conclusions

The study demonstrated the utility of media monitoring for the EMA in the case of the EU referral procedure for HPV vaccines. The presentation of media coverage in the newly developed format of a structured set of derived questions supported scientific assessors and communicators in focussing on specific pieces of information, missing or expected in the public domain, and in enhancing communication proactivity and preparedness. More specifically, the study demonstrated that the derived questions helped the EMA communication department to proactively enrich the public summary statement of PRAC recommendations with medical and methodological aspects. The derived questions further proved ‘ready-for-use’ by the EMA communication department when drafting the talking points for communication preparedness, enabling prompt responses from the EMA at the press briefing and to written queries from journalists. The study demonstrated full predictive capacity regarding the queries from journalists, although in some instances the level of detail of the questions was not anticipated. In addition, the findings from the media monitoring altered the tone of the public statement with acknowledgement of the health status of CRSP and POTS patients, in order to meet public expectations of indicating respect towards patients.

This study seems to be the first on setting up medicinal product-specific media monitoring as a feasible process within a regulatory body in a real-life scenario and evaluating whether it is useful for improving its communication. The study suggests that efficient strategies for medicinal product-specific prospective real-time media monitoring could be part of safety surveillance for medicines of high public health impact and/or high public interest and support not only communication in terms of proactivity and preparedness, but also in relation to transparency and public participation activities. Overall, medicine safety and public trust into underlying surveillance systems could benefit from listening more systematically to the public, identifying and challenging misleading or missing information and addressing what different population groups may want and need to know for making informed choices and using medicines safely and effectively.

## Additional file

Additional file 1: Media outlet sources of articles collected through applying the described search strategy. (DOCX 69 kb)

## Abbreviations

ADVANCE: Accelerated Development of VAccine benefit-risk Collaboration in Europe
CFS: Chronic fatigue syndrome
CRPS: Complex regional pain syndrome
EMA: European Medicines Agency
EU: European Union
HPV: Human papillomavirus
POTS: Postural orthostatic tachycardia syndrome
PPP: Public-private partnership
PRAC: Pharmacovigilance Risk Assessment Committee (of the EMA)
