# Supplementary material for: Application of real-time global media monitoring and ‘derived questions’ for enhancing communication by regulatory bodies: the case of human papillomavirus vaccines
Source: BMC Med. 2017 May 2;15:91. doi: 10.1186/s12916-017-0850-4 (PMC5414293; doi:10.1186/s12916-017-0850-4)
Supplement: Additional file 1: — Media outlet sources of articles collected through applying the described search strategy. (DOCX 69 kb) [file 12916_2017_850_MOESM1_ESM.docx]

**Additional file 1: Media outlet sources of articles collected through applying the described search strategy**

| 1010 WCSI |
| --- |
| 1031 Virgin Radio Winnipeg |
| 104 7 Mix FM |
| 106.9 FM |
| 14 News |
| 1EarthUnited |
| 20 Minutes (France) |
| 20 Minutos (Spain) |
| 24 Hodin |
| 24 News (Canada) |
| 24h Sante |
| 24matins.fr |
| 2MinuteMedicine.com |
| 35 WSEE |
| 4 Nieuws.nl |
| 660 News |
| 680 News |
| 6toPoder |
| 7 KPLC |
| 7thSpace Interactive |
| 91.5 KIOS-FM |
| 96.4 The Wave |
| 9ija News.com |
| A Breaking News |
| A Geeky Girls Blog |
| A Helicopter Mom |
| A Man of no Importance. |
| Aamulehti |
| ABC (Australian Broadcasting Corporation) - Washington DC Bureau |
| ABC 12 News at 5 - WJRT-TV |
| ABC.es Agencias |
| ABCMontana.com |
| Abitare Online |
| About.com Pediatrics |
| AboutPharma |
| ACS News |
| Acta Sanitaria |
| ActionforME |
| Activist Post |
| Actualidad - Famma |
| AD Algemeen Dagblad - Online |
| Advance for Administrators of the Laboratory Online |
| Advocate Health Care Health enews |
| Affaritaliani.it |
| Africa News |
| AfricaNewsDesk.com |
| After the Shift |
| Aftonbladet |
| AG Adjacent Government |
| Age of Autism |
| Agenparl.com (Italy) |
| Agir - Agenzia Giornalistica Repubblica |
| agrealuchadoras.blogspot.com |
| AIDSinfo.gov |
| Aktiespararen |
| Aktiv-Verzeichnis.de |
| Al Jazeera America |
| Al Pueblo Pan y Circo |
| Alabama Public Radio - Online |
| Alands radio |
| Alaska Highway News Online |
| Alaska Native News |
| Albawaba.com |
| Albuquerque Journal Online |
| All News - MPR News |
| allAfrica.com |
| Allodocteurs.fr |
| AllPR.de |
| AlMomento.mx |
| aLome |
| AlphaGalileo |
| Alter Info |
| Altinget.dk |
| Amanpoiare blogspot |
| American Academy of Pediatrics |
| American Cancer Society |
| American Council On Science & Health |
| American Journal of Managed Care Online |
| American Journal of Obstetrics & Gynecology - Online |
| American Journal of Public Health |
| American Pharmacists Association Foundation |
| AMHC |
| Amplify |
| Analyse + Aktion |
| Andina |
| Angers Maville |
| Annonsbladet |
| Ansm.sante.fr |
| Antivakcina.org |
| APM Health Europe |
| [apodyoopsis.tumblr.com](http://apodyoopsis.tumblr.com/) |
| Aporrea.org |
| Apotheke Adhoc |
| ApothekersNieuws.nl |
| Aragón Digital |
| Arbetarbladet |
| ArcaMax |
| Archives Ouvertes - HAL |
| Århus Stiftstidende- Online |
| ARIEGENEWS.COM |
| Arras Maville |
| Art de Vivre Sain |
| Articlesbase |
| Artsenauto.nl |
| ÄrzteZeitung Online |
| Asahi Shimbun (English Edition) - Online |
| Asbury Park Press Online |
| Asiaone.com |
| Aspen Public Radio |
| Astradrom |
| Atlanta Daily World - Online |
| Attitude - Online |
| Australasian Dental Practice |
| Australian Teacher Magazine |
| Avisen.dk - Online |
| Awakestate |
| BAMA - Online |
| BAON - Online |
| Bara jag |
| Barbados Advocate - Online |
| Barometern, OT - Online |
| Bautrend |
| BBC News Online |
| BBC Radio Humberside |
| BBC Radio Wales/Cymru |
| BBC World Service |
| Beaumont Enterprise Online |
| Beauty South Africa |
| Beaver County Times Online |
| Before It's News |
| Belga Press Agency |
| belye nochi blog |
| Bendijin.net |
| Benzinga |
| Beol.hu |
| Berlingske |
| BERLINGSKE BUSINESS - Online |
| Bernardinai.lt |
| Bethune Maville |
| BFM TV - Online |
| BHSkin Dermatology |
| Bhutan Broadcasting Service |
| Biermann Medizin |
| Big Dog 100.9 |
| Billion Toddler March for Survival |
| Bio Pharma Reporter |
| Bioethics.net |
| BioMed Central |
| Biomedical |
| Bio-Medicine |
| BioNews-TX |
| bionity.com |
| BioPharma-Reporter.com |
| BioPortfolio |
| BioSpace |
| BioSpectrum Asia - Online |
| BioTech News International |
| Biotechfinances |
| Bite magazine |
| Blackburn News |
| BlackPlanet.com |
| Blade Online |
| Blasting News United States |
| Blik Op Nieuws |
| Blikk.hu |
| Blog del CENTRO CLÍNICO BETANZOS 60 |
| Blog do Gari Martins da Cachoeira |
| Blog Miguel Jara medicamentos salud industria farmacéutica |
| BLOG SUNTRANEP |
| Bloomberg News - Online |
| Blue town Mountain Tech |
| BN DeStem - Online |
| BNS Spaudos Centras |
| Bob's Blog |
| Bohusläningen - Online |
| Boise State Public Radio |
| Boness Journal - Online |
| BootsWebMD |
| Borås Tidning - Online |
| Bors och Ekonomi |
| Børsen - Online |
| Borsod Online |
| Boston Globe Online |
| Boston.com |
| Bottom Line Secrets |
| Boulevard Voltaire |
| Boursorama.com |
| Brabants Dagblad Online |
| Bradenton Herald Online |
| Brattleboro Reformer - Online |
| Breaking News.ie |
| Brest Maville |
| Bridge River Lillooet News - Online |
| Brief Report (United Kingdom) |
| BrightSurf.com |
| Brignoles Maville |
| Bringing Health Information to the Community (BHIC) |
| Bristol-Warren Patch |
| British Journal of Cancer |
| British Journal of Sports Medicine |
| British Medical Journal - Online |
| Bröllopscoach.se - en blogg om bröllop |
| Buanews |
| Buchan Observer - Online |
| Buga |
| Bundesamt für Gesundheit |
| Bürstädter Zeitung online |
| Business Day Online |
| Business Insider Australia |
| Business Insider India |
| Business Insider UK |
| Business Press24 |
| BusinessReport |
| Butterflies and Wheels |
| Byron Shire News |
| CablePulse 24 (CP24) - Online |
| CADUCEE.NET |
| Caen Maville |
| Cagnes Maville |
| Calgary Herald Online |
| Canada Journal |
| Canada.com |
| Canadian Cancer Society |
| Canadian Medical Association Journal |
| Canadian Online Health News |
| Cancer Epidemiology, Biomarkers & Prevention - Online |
| Cancer News Today |
| Cancer Prevention Research |
| Cancer Research - Online |
| Cancer Therapy Advisor |
| Cancer.dk |
| Cancer.gov |
| CancerCompass |
| CancerNetwork |
| Candace Rose |
| CANOE |
| Cape Breton Post - Online |
| Cape Times - Online |
| CapeNews.net |
| Capital Public Radio - Online |
| Care2 |
| CAREVOX |
| carlorienzi.it |
| Carlow Nationalist |
| Carluke Today - Online |
| Carrick Gazette & Girvan News - Online |
| Catamarcaya |
| Catholic.net |
| CBC News Online |
| CBC Television Network Online |
| CBG-MEB.nl |
| CDC - Centers for Disease Control and Prevention |
| CelebCafe.org |
| Celebrity Diagnosis |
| Celtipharm - Online |
| Central de Noticias blogspot |
| Centre Daily Times Online |
| CFRA-AM Online |
| Chain Drug Review Online |
| Channel 3 News Morning - WCAX-TV |
| Charleston City Paper Online |
| Charlotte Post Online |
| CHAY 9301 |
| C-Health |
| CheckOrphan |
| Cherbourg Maville |
| Chicago Health Online |
| Chicago Tribune Online |
| Chicks with Balls by Judy Takács blog |
| Chimp Reports |
| CHMP-FM Online |
| Cholet Maville |
| CHRE-FM Online |
| ChrisD |
| CHRISTIAN TODAY |
| Chronicle Pharmabiz Online |
| Chronimed |
| CHVR-FM Online |
| CiaComo |
| CIDRAP |
| CIENCIASMEDICASNEWS |
| Cilento Notizie |
| Cilento.it |
| Cittàdella Spezia |
| Citybizlist - Charlotte/Raleigh |
| CIWW-AM (1310 News) - Online |
| CJAD-AM Online |
| CJBK-AM Online |
| CJBX-FM Online |
| CJCS-AM Online |
| CJME RSS |
| CJOB-AM Online |
| CJSB 104.5 |
| CKFR-AM Online |
| CKGL-AM Online |
| CKMM-FM Online |
| CKOM-AM Online |
| CKTB-AM Online |
| CKX-FM Online |
| Clarksville Online |
| clicMedicina |
| Clinical Infectious Diseases |
| Clinical Oncology News - Online |
| Clinician Reviews - Online |
| CLM24.es |
| Clovis News Journal Online |
| Clyde 1 - Online |
| Clyde 2 - Online |
| CNBC Online |
| CNBC World (Squawk Box Asia) |
| CNW Group - Online |
| Coast Guard - Online |
| Coast Reporter - Online |
| Collective Evolution |
| Columbus Dispatch Online |
| Con Salud |
| Concord Monitor Online |
| Concordia University |
| Connecticut Post Online |
| Consultant Online |
| ConsultantLive |
| Contacto Hoy |
| Corporate News |
| Correio Online |
| Correo - Chiclayo - Online |
| Correodelorinoco |
| CorrientesHoy |
| Corriere della Sera - Online |
| COSMiQ |
| COSMOPOLITAN - Online |
| Cosmopolitan Philippines - Online |
| Cote Famille |
| Counsel & Heal |
| Coutinho |
| CPH Post - Online |
| CPhI.cn |
| Craig Daily Press - Online |
| Creatingeve.biz |
| Crosswater Job Guide |
| CTV Montreal - Online |
| CTV News Channel Online |
| CTV Television Network Online |
| CTV Vancouver Island |
| Culture - DCMS |
| Cyber Actu |
| D Gary Grady |
| Dagbladet Holstebro Struer - Online |
| Dagbladet Information |
| Dagelijksestandaard |
| Dagens Industri |
| Dagens Medicin (Denmark) |
| Dagens Medicin (Sweden) |
| Dagens Medisin (Norway) |
| Dagens Nyheter - Online |
| Dagens Pharma |
| dagens.dk |
| Dagensapotek.se |
| Daily Democrat - Online |
| Daily Herald |
| Daily Infographic |
| Daily Journal - Online |
| Daily Monitor - Online |
| Daily Nation - Online |
| Daily Post - Wales - Online |
| Daily Queer News |
| Daily Read List |
| Daily Reporter |
| Daily Vitamina |
| DailyMail Org |
| DailyMe |
| DailyNews724.com |
| dailyRx |
| Dala-Demokraten - Online |
| dalje.com |
| Dallas Morning News - Online |
| Dallas Sun |
| DangoteNews.com |
| Darpan Magazine |
| David Icke |
| Dayton Daily News - Online |
| DC Medical Malpractice & Patient Safety Blog |
| De Gelderlander - Online |
| De Gooi- en Eemlander Online |
| DE LA MEDECINE GENERALE |
| de Nature Herbal |
| De Stentor - Online |
| Deals on Mobile Phones |
| Deccan Chronicle - Online |
| Decode the information |
| Dehir.hu |
| Delawareonline.com |
| Delmagyar.hu |
| Delmarva Life - WBOC-TV |
| Demanjo |
| denik.cz |
| Denmark News.net |
| Denoffentlige |
| Dental Republic - The Probe |
| Dental Tribune - Online |
| Dentistry (United Kingdom) |
| Dentistry Scotland - Online |
| Der Hausarzt |
| Der Nordschleswiger - Online |
| Derma.de |
| desertofsnow blog |
| Deutsches Ärzteblatt |
| Diagnosis Glitter |
| Diagnostic Imaging |
| Diario Amanecer De México |
| Diario Caribazo |
| Diario Chaco |
| Diario de Burgos Online |
| Diario de Pontevedra |
| Diario del Web |
| Diario Dom |
| Diario el Martinense |
| Diario Huarpe |
| Diário Indústria & Comércio |
| Diario La Talacha |
| Diario Los Andes - Perú |
| Diario Marca.com.mx |
| Diario Rotativo |
| Diario Vasco |
| Diariofarma |
| Diariogetafe.diariomadrid.eu |
| Diarionecochea |
| Die Welt der medizinischen Blogs |
| Digital Journal |
| DiLei |
| Dinan Maville |
| DIRECT MATIN - Online |
| Dirigentes Digital |
| Discapnet |
| Discover Paris Tennessee |
| Dknyt.dk |
| Doctor Tipster |
| doctorksa.com |
| Doctors Lounge |
| Doha Insight |
| Dominion Post |
| DomTomNews |
| Don't trade our lives away |
| Douai Maville |
| DR TV - Online |
| Dr. Leonard Coldwell.com |
| Dr. Marc Micozzi |
| Dr. Steve Vasilev \| Integrative Health, Wellbeing and Life |
| Dr. Walt's Health Blog |
| Drag Plus |
| Draguignan Maville |
| Drimble |
| Drug Discovery World (United States) |
| Drug Topics Online |
| Drugging Children |
| Drugs.com |
| Drugzinfo blog |
| Dt.se |
| Dublin News |
| Dublinmick's Breaking News |
| Dxy.cn |
| Dziecko Onet |
| E Medicina |
| e! Science News |
| EastBayRI.com |
| eCanadaNOW |
| eCancer |
| Echo online |
| ED Eindhovens Dagblad Online |
| EDGE Chicago |
| EDGE Dallas |
| EDGE Fire Island |
| EDGE Los Angeles |
| EDGE Medianetwork |
| EDGE Miami |
| EDGE New York City |
| EDGE Palm Springs |
| EDGE Providence |
| EDGE Washington |
| EDI Schweiz |
| Edmonton Journal - Online |
| Edmonton Sun - Online |
| EDP24 |
| Education Week Online |
| Educodomi |
| EHE & me |
| EIN News |
| EIN News Publications |
| Ekstrabladet |
| Ekuriren |
| El Arsenal / Diario Digital |
| El Bravo El Periodico De Tamaulipas |
| EL BRAVO.mx |
| El cinco |
| EL COMERCIO |
| El Correo Gallego - Online |
| El Correo Online |
| El Cronista Diario.com |
| El Debate |
| El Día Online |
| El Diario De Coahuila |
| El Diario de Tuxpan |
| EL DIARIO DE YUCATAN |
| El Diario del Otún |
| El Diario Montañés - Online |
| El Digital de Castilla la Mancha |
| El Economista - Online |
| El Espectador |
| EL GLOBAL - Online |
| El Golfo.info |
| El Gráfico - Online |
| El Informador - Online |
| El Litoral - Online |
| El Manana - Online |
| El Mañana De Valles |
| EL MUNDO - Online |
| El Mundo de Córdoba - Online |
| El Mundo de Orizaba - Online |
| El Mundo-La Crónica de León - Online |
| El Nacional - Online |
| El Norte de Castilla Online |
| El Observador Mexico |
| El Paso Times |
| El Periodico De Aragon - Online |
| El Progreso Online |
| El Siglo de Torreón - Online |
| El Sol de Cordoba - Online |
| El Sol de Cualtla |
| El Sol de Guadalajara - Online |
| El Sol De Mexico Online |
| El Sol de San Luis |
| El Sol de Tlaxcala - Online |
| El Tabloide |
| El Territorio del NEA |
| El Tiempo (Argentina) - Online |
| El Tiempo (Colombia) - Online |
| El Universal (Colombia) - Online |
| El Universal Online |
| elColombiano.com |
| Eleftheria.gr |
| Elespectador.com |
| Elgraficotam.com.mx |
| Eliseeva blogspot |
| Elizabeth Women's Clinic |
| ELLE - Online |
| ElRegio.com |
| eluniversal.com |
| Elvigia.net |
| EM-consulte |
| EnergeticCity.ca |
| Enews.Shafaqna.com |
| ENEWSPAPER |
| Englemed Health News |
| Enjoy Your Remaining Years! |
| EnLíneaDirecta.info |
| Enterese.net |
| ENTRE VEREDAS |
| ENVIRONMENTAL ILLNESS NETWORK |
| Epoznan.pl |
| Equities.com |
| E-sante.be |
| E-sante.fr |
| Essential Baby |
| están cambiando los tiempos |
| EurakeSante.fr |
| EurekAlert |
| Europa Press Online |
| Europe 1 - Online |
| European Commission |
| European Pharmaceutical Review - Online |
| EuroSurveillance |
| Evansville Courier & Press Online |
| Evening News 24 |
| Everyday Devotional |
| Everyday Health |
| Everyone's Blog Posts - peacepink |
| EXAME.com |
| Excélsior |
| Exchange Online |
| exilapotekare |
| Expatica France |
| Expertsvar |
| Expreso.com.mx |
| Expreso.press |
| Express (United Kingdom) |
| Expressen - Online (Sweden) |
| Extraterrestrials.ning.com |
| FABERGE GOOGLE DOODLE |
| Face The State - KTVN-TV |
| Fact Not Fiction |
| Facts (Switzerland) |
| FAGG (Belgium) |
| Familie Journal (Denmark) |
| Family Survival Protocol - Microcosm News |
| Famma-Cocemfe Madrid |
| FARMA Y SALUD |
| FARMACOSEGURIDAD |
| Faro de Vigo Online |
| Fawkes News |
| FDA News |
| FDA Webview |
| Federfarma.it |
| Femin'Actu |
| Femme actuelle - Online |
| Fern Avenue Public School |
| FiercePharma Marketing |
| FiercePracticeManagement |
| FierceVaccines |
| Fimea |
| finanzen.at |
| Finanzen.ch |
| Fingal Independent - Online |
| First Read |
| Flurry of Thoughts |
| FM 90.5 Peterborough |
| FMT Gezondheidszorg |
| Focus Taiwan |
| Folha da Manhã Online |
| Folkbladet.se |
| Folkebladetlemvig |
| Folketinget |
| Folkhalsomyndigheten |
| Foodconsumer.org |
| FoodWorldNews.com |
| ForceChange |
| ForeignAffairs |
| Forfar Today - Online |
| Formare Medicala |
| Forskning.se |
| Fort Collins Coloradoan Online |
| Forth 1 |
| Forum Online |
| Fox 13 News at 10 - WTVT-TV |
| FOX 45 News at 4:00 - WBFF-TV |
| Fox 5 News at 5 - WNYW-TV |
| Fox 59 News at 4 - WXIN-TV |
| Fox 8 News at Noon - WVUE-TV |
| FOX News Channel Online |
| Fox News Special: Your Health - WTXF-PHI (FOX) |
| FOX40 News at 4:30 AM - KTXL-TV |
| FOX6 WBRC - MyFoxAL.com - News |
| Fox8 News at 5:00 AM - WGHP-TV |
| France 24 - Online |
| France Bleu - Online |
| France Inter |
| Franceinfo.fr |
| France-Politique.over-blog.com |
| FRANCETV INFO |
| Frauenärzte im Netz |
| Fredericia Dagblad |
| Fredericia Dagblad - Online |
| Free - Online (France) |
| Freedom Fighters of America |
| Fresh News |
| Fresno Bee Online |
| From the Trenches World Report |
| FronteraEnsenada.info |
| Frontshop Pharmacy Magazine |
| FT - Gavyn Davies |
| FT.com |
| Fyens Stiftstidende |
| G1 |
| Gaceta Medica.com |
| gacetademexico.com |
| Galloway Gazette - Online |
| Gay News Network |
| Gay Times magazine |
| Gayapolis |
| GAYSTARNEWS |
| GAZET VAN ANTWERPEN |
| Gazeta.pl |
| GDPUK |
| Genova24.it |
| Gente Online |
| Georgia Public Radio - Online |
| GESTIÓN EN SALUD PÚBLICA |
| Gestiona Tudinero |
| Gesundheitsspiegel \| Das Blog rund um ihre Gesundheit |
| Gesundheitsstadt Berlin |
| Gezondheid bog |
| Gezondheid.be |
| gilmagamezPeriodista |
| Giornalettismo |
| GirlPower.it |
| Glam Touch |
| Glasgow South and Eastwood Extra |
| Global News |
| Global Warming & Terra Forming Terra |
| GlobeAdvisor.com |
| Gloria.tv - The more catholic the better |
| Glowbi |
| Go Health Insurance Blog |
| Go Salute |
| GodfreyDaily |
| Godlike Productions - Pinned Threads |
| Golden Age of Gaia |
| Goshen News Online |
| Goteborgs-Posten |
| GOVERNMENT SLAVES.INFO (USA) |
| GP Online |
| Grantham Journal & Citizen - Online |
| Graphic Ghana |
| Graphic Online |
| Great Bend Tribune Online |
| Greenfield Daily Reporter - Online |
| GreenMedInfo |
| Greenwich Time Online |
| GRENZECHO.net |
| gretler.com |
| Grupo Fórmula |
| Grupo Gamma |
| Grupo Noreste - Online |
| Grupo Radio Gandia |
| Gscene |
| GT (Online) |
| Guernsey Press |
| Guía Médica, consejos de Salud |
| Guide & Gazette |
| Gulf Times - Online |
| Guyana Chronicle - Online |
| Guyana Times |
| GWMS News |
| Hafens Hörna |
| HAITI RECTO VERSO |
| Hajdú Online |
| Hallands Nyheter |
| Hallandspostan |
| Ham & High Broadway - Online |
| Hamburger Abendblatt - Online |
| Hans Gruen |
| Haravgi - Online |
| Harvard Health Letter |
| Harvard Medical School |
| Hawaii News Now |
| Hawick News |
| HáziPatika.com |
| HB Informação |
| Headlines 24 (Netherlands) |
| Headlines India |
| Headlinez.nl |
| HealingWell.com |
| Healio.com |
| Health & Love Page \| RSS Feed |
| Health Canada |
| Health Impact News |
| Health News |
| Health On the Net Foundation |
| Health Promotion Practice current issue |
| Health Supreme \| Scoop.it |
| Health, Medical, and Science Updates |
| Health.com |
| Health.com communities |
| Health24 |
| HealthCanal.com |
| Healthcare Quarterly - Longwoods - Online |
| HealthDay |
| HealthDesk |
| HealthIT Analytics |
| Healthline |
| Healthyyou-intheair blog |
| Heilbronner Stimme Online |
| helagotland.se |
| Helse |
| Helse & Fitness |
| Helsingborgs Dagblad - Online |
| Hemophilia is for Girls |
| Heraldo de Aragón Online |
| HERALDO SANITARIO DE OREGÓN |
| Herald-Times Online |
| herenciageneticayenfermedad |
| Herning Folkeblad |
| Herpes Survival Kit |
| HET BELANG VAN LIMBURG (hbvl.be) |
| Heute.at |
| Hiding in Plain Sight |
| Hidrocalidodigital.com |
| Hillary Chybinski |
| Hindu Business Line Online |
| Hirado.hu |
| Hoje em dia Online |
| Holy Hormones |
| Honolulu Star-Advertiser Online |
| HORACERO |
| Horsens Folkeblad |
| Hospital Pharmacy Europe |
| Houses of the Oireachtas |
| Houston Northwest Medical Center |
| How the hell should I know? |
| Hoy Diario de Extremadura - Online |
| HQ Prince George |
| HT Syndication |
| Huckabee Online - FOX News Channel |
| Hudson Valley News Network |
| Huffington Post Canada, The |
| Huisartsvandaag |
| humboldtjournal.ca |
| Humeurs de Marissé |
| I Knew This Would Happen! |
| iAfrica.com |
| Idaho Statesman Online |
| Ideal Online |
| iDiva |
| iDNES.cz |
| iFreePress |
| iHealthBeat |
| Il Farmacista |
| Il Nazionale |
| Il Sole 24 Ore Online |
| Il Tempo - Online |
| Il Terreno |
| ilcentro.gelocal.it |
| Ilford Recorder Series - Online |
| Ilkka |
| Immunisation Horizon Scanning |
| Index - Online |
| Individual.com |
| Infection Control Today Online |
| Infectious Disease |
| InfoDocket |
| Inforadio |
| Information Online |
| Information Radiologue |
| INFORMAZIONE OLTRE CENSURA blog |
| Infosalus |
| InfoTel |
| In-PharmaTechnologist |
| Inquisitr |
| Inside Philanthropy |
| Insurance News Net Online |
| Inte bara sjuk |
| Interlochen Public Radio |
| International AntiCounterfeiting Coalition |
| International Business Times AU |
| International Business TImes Australia - Online |
| Intervalolibre |
| Inverurie Today - Online |
| IOL.co.za |
| Ionia Sentinel Standard Online |
| Iowa Public Radio - Online |
| Irish Examiner - Online |
| Irish Independent |
| Irish Medical Times |
| Irish Sun |
| IrishExaminer.com - Ireland |
| iSanté |
| Israel Herald |
| Isthmus Online |
| it.notizie.yahoo.com |
| IT-BUSINESS Online |
| IT-Nytt.nu |
| ITV Network Ltd - Online |
| iX-Magazin für professionelle Informationstechnik |
| Jaclyn's Cookies |
| Jama pediatrics |
| Jamaica Observer - Online, The |
| jarek-kefir.org |
| JDreport.com |
| Jefferson Public Radio Network Online |
| Jersey Evening Post - Online |
| Jiji Press America Online |
| JIM.FR |
| Jornal da Manhã - Online |
| Jornal O Expresso |
| Journal & Courier - Online |
| Journal de Saône et Loire - Online |
| Journal des Femmes : Derniers contenus |
| Journal ES Hoje |
| Journal of Clinical Oncology - Online |
| Journal of Forecasting |
| Journal of the American Board of Family Medicine Online |
| Journal of the American Pharmacists Association - Online |
| Journal Watch |
| Journalisten.se |
| Journal-Pioneer Online |
| Jugo |
| JydskeVestkysten |
| Jyllands-Posten - Online |
| K93 |
| Ka Leo O Hawai'I - Online |
| Kainuun Sanomat - Online |
| Kaiser Permanente |
| Kaksplus - Online |
| KALW |
| Kankakee Valley Post-News - Online |
| Kansas Public Radio |
| KARE-TV Online |
| Karjalainen - Online |
| KASU |
| Katrineholms-Kuriren |
| KBIA-FM - Online |
| KCBD |
| KCBX-FM - KCBX-FM online Bureau |
| KCCU |
| KCEN HD |
| Kcen TV |
| KCLR 96FM |
| KCUR-FM Online |
| Keerzijde.org Nieuws |
| kelsium.tumblr.com blog |
| Kempton Express |
| KERA news |
| KERA think |
| KFBB-TV - Online |
| KFDA-TV Online |
| Kfm Radio |
| KFMB-FM (Jack FM) - Online |
| KFVE-TV Online |
| KHJ.ca |
| KHQ Right Now |
| Kildare Nationalist |
| Kincardineshire Observer - Online |
| Kinder- u. Jugendärzte im Netz online |
| KING 5 News at 11 - KING-TV |
| King World News |
| KIRO-AM |
| Kirriemuir Herald - Online |
| kisalfold.hu |
| Kiwiblog |
| KJZZ-FM - Online |
| KKOH-AM |
| KLCC-FM - Online |
| KLST News at 6:00 PM - KLST-TV |
| Klubrádió |
| KMIR-TV - Online |
| KMOV-TV - Online |
| KNBA-FM - Online |
| Know Your "V" - blogspot |
| KNOW-FM |
| Knowledge of Medicine |
| KNPR-FM (Nevada Public Radio) - Online |
| KOAA-TV Online |
| KochaneZdrowie |
| Kolding |
| Koninklijke Nederlandse Maatschappij ter bevordering der Pharmacie(KNMP) |
| KPBS-TV - Online |
| KPCC-FM - Online |
| KPLC TV |
| KPLU-FM - Online |
| KPRC Channel 2 News Midday - KPRC-TV |
| KQED-FM - Online |
| Kræftens Bekæmpelse |
| Krankenhaus.net |
| Krankenpflege journal |
| KRCB |
| Kristeligt Dagblad - Online |
| Kristianstadsbladet - Online |
| KRIS-TV - Online |
| KRQE-TV - Online |
| KRVS-FM - Online |
| KRWG-FM - Online |
| KSAN 3 News at 10 PM - KSAN-TV |
| KSDK-TV - Online |
| KSL 5 News at 5 - KSL-TV |
| KSL-TV - Online |
| KSMU-FM - Online |
| KSTS-TV - Online |
| KSWO-TV |
| KTBS 3 |
| KTEN.com |
| KTIV-TV - Online |
| KTTC-TV - Online |
| Kuam |
| kuar.org |
| KULR-TV - Online |
| KUNM |
| KUNR-FM (Reno Public Radio) - Online |
| KUOW-FM - Online |
| KVCR-FM - Online |
| KWES NewsWest 9 |
| kwgs.org |
| KWWL-TV - Online |
| KXLN-TV |
| KXLT-TV - Online |
| KXXV-TV |
| Kyrkans Tidning - Online |
| KYW-TV - Online |
| L' actualite - Online |
| L' Huffington Post Italia |
| La Cerca - Online |
| LA CROIX - ONLINE |
| La Crónica - Online |
| La Crónica de Guadalajara |
| La Dépêche du Midi - Online |
| La Gaceta (Ecuador) |
| La Informacion |
| La Información República Dominicana |
| La Jornada - Online |
| La Leva di Archimede (ENG) |
| La Mañana de Córdoba - Online |
| La Manche Libre - Online |
| La Nacion (Argentina) - Online |
| La Nación Dominicana |
| La Nouvelle Republique (France) - Online |
| La Opinión - Online |
| La Opinión A Coruña - Online |
| La Opinión de Málaga |
| La Patria - Online |
| La Prensa (Bolivia) - Online |
| La Prensa (Honduras) - Online |
| La Primera |
| La Provence - Online |
| La Razón |
| La Repubblica - Online |
| La Rioja - Online |
| La rioja news |
| La Tibuna de Toledo - Online |
| La Verdad - Alicante - Online |
| La Verdad del Sureste - Online |
| La Voz Digital |
| Laegerudensponser |
| laegerudensponser.wordpress.com |
| Läkartidningen |
| Lake forest health and fitness |
| LäkemedelsVärlden |
| Lakemedelsverkets |
| Lamontagne.fr - Online |
| Länstidningen Södertälje - Online |
| Lao Dong - Online |
| Lareb.nl |
| Las Provincias Online |
| Lasalud.mx |
| Latest Nigerian News |
| Latining |
| Latinos Health |
| L'avenir.net |
| laVoceIdealista |
| Lawyer Press (Spain) |
| LCI.fr - Online |
| Le Bien Public/ Les Dépêches - Online |
| Le blog de hugo |
| Le Devoir Online |
| Le Figaro - Online |
| Le fil d'actualité de service-public.fr |
| Le Generaliste |
| LE HUFFINGTON POST |
| LE JOURNAL DES FEMMES |
| Le Journal du Centre - Online |
| Le Journal Du Dimanche - Online |
| Le Journal Saint-Francois |
| Le Lézard.com |
| Le Monde.fr |
| Le Nouveau Paradigme |
| Le Parisien - Aujourd'hui en France - Online |
| Le Point.fr |
| Le Quitiden du Medicin |
| Le Republicain Lorrain - Online |
| Le site d'Initiative Citoyenne |
| Le Télégramme - Online |
| Lebabi |
| Leberry.fr - Online |
| Lefigaro.fr feeds |
| Lepopulaire.fr |
| Les derniers articles publiés sur Meryl Yourish - Centerblog |
| Les derniers commentaires sur le blog islamsunna.centerblog.net |
| Les Echos - Online |
| Les moutons enragés |
| Lethbridge Herald |
| L'express - Online |
| LGBT Bristol |
| Liberación AHORA |
| Libération - Online |
| Libero 24x7 |
| Lietuve |
| Life |
| Like To Be Fit |
| LINFO.re |
| Linkeol, L'actualité des entreprises en France |
| Linlithgow Gazette |
| Little Green Footballs |
| LJ INFOdocket |
| LOADEER |
| L'OBS - Online |
| Local Denmark |
| LocalNews7.com |
| LocalUKNews.co.uk |
| Lokalavisen Norddjurs |
| Lokalavisen Sønderborg |
| Lokalavisen.dk |
| Lokalavisen/Grenaa |
| Lolland-Falsters Folketidende - Online |
| Lommelegen.no |
| London School of Hygiene & Tropical Medicine |
| Loop Barbados |
| Lørdagsavisen |
| Lordskelradath.tumblr blog |
| Los Andes |
| Los Angeles Metro Bugle |
| Love, Lust & Life blog |
| ltz.se |
| LWW Journals |
| L'Yonne Républicaine - Online |
| M2 Pharma |
| Ma santé facile |
| ma.hu |
| Madame Noire |
| Magasinet Pleje |
| Magyar Nemzet Online |
| MagZ La ViE |
| Mail & Guardian Online |
| MailOnline |
| Maine Public Broadcasting |
| Mainichi.jp |
| Mamba Online |
| Manchester Evening News (Online) |
| Manchester University |
| Manitoba |
| MARANAUTA blogspot |
| Marco A. Mares |
| Maria Lopes e Temas Transversais |
| Marin Independent Journal Online |
| Marion Star Online |
| Market Realist |
| Marketplace Online |
| Marlborough Express - Online |
| Matlock Mercury & West Derbyshire News - Online |
| Mayamiyazono |
| MayoClinic.com |
| Me and My Doctor |
| Meaner Than My Demons |
| Mearns Leader - Online |
| medbroadcast.com |
| Medgadget |
| Mediacongo |
| Medical and Health Update |
| Medical Daily |
| Medical Economics |
| Medical News Today |
| Medical Practice & Dispensary News |
| Medical Principles and Practice - Online |
| medicalonline.hu |
| MedicalResearch.com |
| MedicalXpress.com |
| Medicin och farmaci |
| Medicina |
| Medicine Hat News - Online |
| MedicineNet.com |
| Medindia.net |
| Medinews.be |
| Mediste.fr |
| Medizin 2000 |
| MedNews |
| medpageTODAY |
| Medscape |
| MedWatch.dk |
| Meh. |
| Meinbezirk.at |
| Men's Health Forum |
| Mensch und Krebs |
| Mercola.com |
| MessiahMews Blogs |
| Metro - Online (Canada) |
| Metro - Online (Sweden) |
| Metro - Stockholm |
| Metro Montreal |
| Metro News Peru |
| Metroland.com |
| Metronoticias |
| metropol.hu |
| Metroxpress (Denmark) - Online |
| MiCiudadReal.es |
| Midi Libre - Online |
| Midland Daily News - Online |
| Midlothian Advertiser - Online |
| Midtown Blogger/Manhattan Valley Follies |
| Milano Online |
| Milano Today (Italy) |
| Milenar - Construindo um novo Amanhã |
| Milenio |
| MilitarySpot - Online |
| Milngavie Herald - Online |
| Milwaukee Community Journal Online |
| Minap.hu |
| Ministère de la Santé et des Services Sociaux |
| Ministeriet for Sundhed og Forebyggelse |
| Minyanville |
| Mirror - Online |
| Misiones - Online |
| Miss Eco Glam |
| Miss.at |
| Mississippi News Now |
| Missoula - Bozeman |
| Mittelstand Cafe |
| mja.dk |
| Mobile WebMD Health |
| ModernMedicine.com |
| Moderný svet |
| Mom.me |
| MOMmunizations |
| Monaco-Matin |
| Monat |
| Mondaq |
| Money News |
| MoneyShow.com |
| Montana Public Radio |
| Monthly Prescribing Reference Online |
| Montreal Gazette |
| Montrealexpress - Online |
| Montrose Review - Online |
| Moray Firth Radio - Online |
| Morningstar.ca |
| Moteris.lt |
| Motherwell Times Series - Online |
| Mountain View Telegraph |
| MRN News |
| msn (Belgium) |
| msn (Denmark) |
| msn (France) |
| msn (South Africa) |
| msn (Switzerland) |
| msn Sport (Sweden) |
| msn Video (Canada) |
| Multimedia Feed |
| MummyPages |
| Mundillo Politico blog |
| My apple a day blog |
| My East Tex |
| My informs |
| My mess/ms blog |
| My Office Magazine |
| My Secret Atheist Blog |
| myafrica.allafrica.com |
| MyNewsdesk Sweden |
| MyPE News |
| MyRepublica.com |
| mySteinbach.ca |
| Mytoba |
| NA (Sweden)- Online |
| Namibia Press Agency - Online |
| National Board of Health (Denmark) |
| National Cancer Institute (USA) |
| National Newswatch |
| National Post Online |
| Nationale Zorggids |
| Natur – fältbiologi – naturvård |
| Natural Solutions Radio |
| NaturalNews.com |
| NBC 10 News at 5:30pm - WJAR-TV |
| NBC 10 News Sunrise at 4:30am - WJAR-TV |
| NBC12 - Online |
| nbjour |
| NE10 |
| Nebraska Medicine |
| NECN/New England Cable News Online |
| NEJM Journal Watch: Physician's First Watch RSS Feed |
| NET (Nebraska's PBS & NPR Stations) - Online |
| netdoktor.at |
| NetDoktor.se |
| Netzfrauen |
| New England Public Radio |
| New Haven Register |
| New Jersey Herald - Online |
| New Pittsburgh Courier - Online |
| New Straits Times - Online |
| New Vision - Online |
| New York Online |
| New York Parenting |
| New York Times |
| New York University |
| New Zealand Herald Online |
| New Zealand News UK |
| NewNowNext |
| Newratings.de |
| News 12 Connecticut |
| News 14 Charlotte |
| News 4 WOAI Today - WOAI-TV |
| News and Tribune - Online |
| News Channel 10 |
| News Deutschland |
| News Informer.info |
| News List Online |
| News Reality |
| News Talk 980 CJME |
| News WECT |
| News West 9 |
| News Works |
| News.dk |
| NewsCenter 7's Nightbeat - WHIO-TV |
| NewsChannel 3 News at 11 - WTKR-TV |
| NewsChannel 6 Now |
| NewsHub (France) |
| NEWS-Line |
| NewsMax (Unites States) |
| Newsmax Online |
| NewsOnFeeds.com |
| NewsR.in |
| NewsRx.com |
| Newstalk |
| Newstalk 650 CKOM |
| News-Times Online |
| Newswise |
| NhatBao |
| NHFT Libraries Daily Health News |
| NHPR/New Hampshire Public Radio Online |
| NHS Wales |
| Niagara Frontier Publications - Online |
| Nice Matin - Online |
| Nice Maville |
| Nieuws.be |
| Nieuwsblad.be |
| Nieuwsmeldingen.nl |
| NigerianHerald.com |
| NIH MedlinePlus Online |
| Nina Says |
| NineForNews |
| NL Times |
| Nok Lapja Cafe |
| NOODLS |
| Noozilla |
| Nordic Business Report |
| Nordjysken |
| Nordvestnyt |
| Norra Skåne - Online |
| Norran |
| Norrbottens-Kuriren |
| Norrköpings Tidningar |
| Norrländska Socialdemokraten |
| Norrtelje Tidning |
| North County Leader (Ireland) |
| Northumberland View - Online |
| Norwegian Institute of Public Health |
| Norwich Evening News 24 |
| NOS.nl |
| Nothing Special |
| Noticanarias- Online |
| Noticias Caracol |
| Noticias de Oaxaca |
| Noticias de Tamaulipas |
| Noticias del Mundo |
| Noticias Galicia |
| Noticias Voz e Imagen |
| Noticias24 |
| Noticieros Televisa |
| Nouvelobs.com RSS |
| NovaNewsNow |
| Novi list - Online |
| Novinky.cz |
| NPInvestor.dk |
| NPR Online |
| NSK |
| nsnbc international |
| NTN24 |
| NTR Zacatecas |
| Nu.nl |
| Nueva Mentes |
| Nujij.nl |
| NurseLucy.com |
| Nursing in Practice |
| NursingTimes.net |
| NUsport.nl |
| NUTesla \| The Informant |
| Nutritional Outlook - Online |
| Ny Medicin |
| Ny Teknik |
| Nya Dagbladet |
| Nyheter (Ekot) |
| Nyheterna |
| NyheterNu.se |
| Nyhetsnotiser |
| O MAN |
| O Tempo - Online |
| Observer-Reporter - Online (Unated States) |
| Oem.com.mx |
| Off The Grid News |
| Offsetting Behaviour |
| OggiTreviso |
| Õhtuleht |
| Okawan |
| ÖKO-TEST Online |
| On Politics (RI NPR) |
| ON REFAIT LES COURSES feedsportal |
| OncoLink |
| Oncology Live Online |
| Oncology Nurse Advisor - Online |
| One News Page (Global) |
| One News Page (United States) |
| Onemillionvelociraptors blog |
| Onet.pl |
| onlinepresse.info |
| ONMEDA |
| OnMedica |
| ONS Connect - Online |
| Opanoticias |
| Oral Cancer News |
| Orange (France) - Online |
| ORF.at |
| Organic Life Online |
| Organizacion Editorial Mexicana - Online |
| OrientPress Hírügynökség |
| OrosCafé |
| Oroville Mercury-Register Online |
| Orthopedics Today - Online |
| Osap.org |
| OsideNews.com |
| Oskaloosa News |
| Österbottens Tidning |
| Östgöta Correspondenten - Online |
| Ostra Smaland |
| OtoRhinoLaryngology - Alexandros G. Sfakianakis |
| Ottawa Citizen Online |
| Ottawa Sun Online |
| Ouest-France - Online |
| OUR WINDSOR |
| Out of Sense |
| Outbreak News Today |
| OutSmart |
| Oxygen.ie |
| P&T Community |
| Padova News |
| Pain Relief blog |
| PAIRSonalities UK |
| Pak Tea House |
| PAPANTLA EN LA NOTICIA (Información plural, real y confiable...) |
| Paperblog (France) |
| Paperblog (Italy) |
| Paperblog (Spain) |
| Papua New Guinea Post-Courier - Online |
| Paradise Post - Online |
| Parent Herald |
| Parents Online |
| Parents.fr |
| Paris Maville |
| Paris-Normandie - Online |
| ParlamentniListy.cz |
| Passio Umbra |
| Patheos |
| PBS Online |
| PCOS Message Board |
| pecsma.hu |
| PEDIATRICS (Unites states) |
| Pediatrics blog (United States) |
| Penticton Herald Online |
| Penzcentrum.hu |
| Peoria Public Radio |
| Periódico Novedades de Tabasco |
| Periódico Victoria.mx |
| Periodicoexpress - Online |
| Periodistas en Español |
| Pernambuco.com |
| Peruinforma.com |
| Pharma Focus Asia - Online |
| Pharmaceutical Business Review |
| Pharmaceutical Processing Online |
| Pharmacie.ma |
| Pharmacy Choice |
| Pharmacy Daily |
| Pharmacy Life News |
| Pharmacy Times Online |
| Pharmacy Today |
| PharmaLive |
| Pharmastar |
| PharmaVOICE - Online |
| Pharmiweb |
| Pharos-Tribune Online |
| Philadelphia Business Journal Online |
| Philadelphia Inquirer Online |
| Philipine canadian inquirer |
| Philly.com |
| Philosophers Stone |
| Philosophical Garbage |
| Phnom Penh Post - Online |
| Physicians Briefing |
| Physicians Weekly |
| PinkNews |
| PINN Online |
| Pipeline Review |
| Pise.cz - vsechny blogy |
| Piteå-Tidningen |
| PLANET.fr |
| Planned Parenthood |
| Plenilunia - Online |
| Plus Magazine- Online (plusmagazine.knack.be) |
| Pluska |
| PM360: The Full Spectrum Of Product Management - Online |
| Pocono Record Online |
| POLITIKEN - Online |
| Politis.com.cy Online |
| Politism |
| Por Esto! |
| Por Trás da Mídia Mundial |
| Portail Free |
| Portail SFR (France) |
| Portal america21.de |
| Portales News-Tribune - Online |
| Potilaan Lääkärilehti |
| Pourquoi Docteur? |
| POZ |
| PR Finder |
| PRACowniA |
| Prairie Public Broadcasting |
| Press & Sun-Bulletin Online |
| Presscontacts.com |
| Presse Box |
| Press-Leader |
| Pressperu.com |
| PressReleasePoint |
| Primary Care Today |
| Primera Hora |
| Primocanale |
| Primorske Novice - Online |
| Prince Albert (Panow.com) |
| Prince George Citizen Online |
| Prince George Free Press |
| Pro & Contra - Online |
| Procuradoria Geral da República - MPF |
| Profit Quotes |
| Proinvestor |
| Projectqueer |
| Propaganda Pre$$ Monitor |
| Propeller |
| Prospect - Online |
| Providence Journal Online, The |
| Provinciale Zeeuwse Courant |
| Prozeny.cz |
| Pta Forum |
| Public Broadcasting - Online |
| Public Health |
| Publimetro México |
| PUEBLA Noticias (Mexico) |
| Pusha - Nya länkar |
| Qatar is Booming |
| Q-Notes |
| Quadratin |
| Questions de femmes |
| Quincy Herald-Whig Online |
| QX (qx.se) |
| R7 |
| rabble.ca |
| Radins.com |
| Radio Canada International - Online |
| Radio Caracol |
| Radio Intereconomia |
| Radio Kerry |
| Radio New Zealand National - Online |
| Radio RSI Rete Uno (Italy) |
| Radio1.pf |
| Radio-Canada Réseau La Première Chaîne Online |
| Randers Amtsavis |
| realitatea.net |
| Redaccion Medica |
| Refinery29 |
| Reforma - Online |
| Reformatorisch Dagblad |
| Regine Leader-Post |
| Regional Alliance for Healthy Schools (RAHS) |
| Regulatory Focus Online |
| Reiki Dawn |
| René Guiart blog |
| Republic Online |
| Republican Journal Online |
| Republika |
| République Togolaise |
| Researching Health |
| Reto Diario |
| Reuters Feedsportal |
| Reuters India |
| Reuters UK |
| Reuters US |
| Revista Gerente |
| Revista Mundo Nuevo |
| Revistalegal.com |
| Rewire |
| RFSL |
| Rhein Main Presse Online |
| Rhode Island Public Radio |
| Rhode Island Public Radio Online |
| Ringkjøbing Amts Dagblad - Online |
| Río Negro Online |
| Risk Factors for... |
| Rocket News |
| Romania TV |
| Romford Recorder Series - Online |
| Roscommon Herald |
| Roskilde Avis |
| RTV Utrecht |
| RxList |
| Rynek Aptek |
| Salud General: informacion y tratamientos |
| Salud180 |
| SaludToday Blog |
| Salute Domani |
| Salute olistica: benessere globale |
| Samashies.tumblr blog |
| San Antonio Express-News Online |
| San Diego Union-Tribune Online |
| San Francisco Chronicle Online |
| Sändaren |
| Sanremonews.it |
| Santa Cruz Sentinel Online |
| Sante blog (blog.santelog.com) |
| Sante Magazine |
| Sascho's Blog |
| Saude Plena |
| Savon Sanomat |
| SavonaNews.it |
| SBWire - Online |
| ScholarlyCommons |
| Science & Vie - Online |
| SCIENCE.NEWZS . de |
| Sciencecodex.com |
| ScienceDaily |
| ScienceNewsline |
| SciLogs (Germany) |
| SciLogs.com |
| Scoop INDEPENDENT NEWS |
| Scoop-santé |
| Scots Auto Scene - Online |
| scotsman.com |
| SCRIP |
| sdpb.org |
| Season Liberally With Wild Falsehoods |
| Secretly Healthy \| RSS Feed |
| Secretos de Salud.es |
| Seeking Alpha |
| Selkirk Weekend Advertiser - Online |
| Semeando |
| Sen Trang blog |
| SEN360 |
| Senator Fidelma Healy Eames site |
| Seulementpourlesamoureux blog |
| Sex And Psychology |
| SGTreport - The Corporate Propaganda Antidote |
| Shannon Side |
| Sheep Media |
| Sheila Kennedy blog |
| Shot of Prevention |
| Shout Your Site |
| Shruglife |
| Siam Longings |
| Sidney Kimmel Cancer Center |
| Siempre889 |
| Sigalon's Soup - made by the Swedish Frog |
| Sigma Live |
| Sin Embargo |
| Sjællandske Medier |
| Skai TV |
| Skaraborgs Lans Tidning |
| Skive Folkeblad/Midt på Ugen - Online |
| SKNVibes |
| Skvallernytt.se |
| Smålandsposten |
| Smile-on |
| Social Dashboard |
| Sociedad de Mejoras Públicas de Manizales |
| Socio-Economics History Blog |
| Södermanlands Nyheter - Online |
| Sofokleousin.gr |
| Someone Somewhere (zedie.wordpress.com) |
| Sonline.hu |
| Sootoday.com |
| SOTN: Alternative News & Commentary |
| South Bend Tribune - Online |
| South Wales Argus - Online |
| Southern Courier - Online |
| Southern Reporter - Online |
| Southern Rhode Island Newspapers - Online |
| Southland Times - Online |
| Spartanburg Herald-Journal - Online |
| Spectrum Health Beat |
| spittingdiamonds |
| Spoke |
| Sporten.dk |
| Spravy.Pozri.sk |
| sprechzimmer.ch |
| Springfield News-Leader Online |
| Springfield News-Sun Online |
| Spy Ghana |
| Squamish Chief - Online |
| SRI - Sistema Radiofónico Informativo |
| St George and Sutherland Shire Leader - Online |
| St. Louis Post-Dispatch Online |
| Stamford Advocate |
| STAR FM 93,3 |
| State Journal - Online |
| Statens legemiddelverket |
| Statens Serum Institut |
| Statesman Journal Online |
| STDs Blog |
| Stiri Rol (Romania) |
| Stockholms läns landsting |
| Stockhouse |
| StockNod |
| STOP.hu |
| Stornoway Today - Online |
| Stranger in a Strange Land |
| Strategy Business Group |
| StreetInsider |
| StreetInsider.com |
| Stuff Health (New Zealand) |
| stuff.co.nz |
| Stuttgarter Zeitung - Online |
| Suburban Press - Online |
| Sud Ouest |
| Sud Radio |
| Sumedico.com |
| Sun Journal - Online |
| Sun Times National |
| SundayWorld |
| Suomen lääkärilehti |
| Super Express Online |
| Suplemento Investigacion y Desarrollo |
| SUR Online |
| Sustainability (Thomson Reuters) |
| Svenska Dagbladet - Online |
| Sveriges Radio |
| Swansea Sound |
| Swedish Television - Online |
| Sydöstran |
| Sydsvenskan - Online |
| Sys-Con Italy |
| Szoljon.hu |
| Szon.hu |
| Tahiti Infos |
| Taka Gazetesi |
| Tandvårdsskadeförbundet |
| Tanta Salute |
| Tatumba.com |
| Teacher Magazine |
| Tech Times |
| Teen VOGUE - Online |
| Televisa |
| Television Nacional de Peru - Online |
| Teraz |
| TermPaperWarehouse |
| Terra Nuova |
| TERRA REAL TIME |
| Terra.com |
| TERrafirmaUSA |
| Terve.fi |
| Texarkana Gazette Online |
| Texas Public Radio - Online |
| TF1 - Online |
| The Argus (Lakehead University) |
| The ASCO Post - Online |
| The Australian - Online |
| The Ballarat Courier - Online |
| The Berwickshire News |
| The Brandon Sun Online |
| The Brown Daily Herald |
| The Bulletin - Online |
| The Calgary Sun Online |
| The Canadian Press Online |
| The Chronicle-Journal Online |
| The Citizen (Tanzania) |
| The Citizens Voice |
| The Cleveland Jewish News Online |
| The Clinical Advisor |
| The Coffs Coast Advocate - Online |
| The Conspiracy Index |
| The Conversation |
| The Cut |
| The CW-38 GA-BAMA |
| The Daily Courier - Online |
| The Daily Dot |
| The Dentist |
| The Elkhart Truth - Online |
| The ELLON TIMES |
| The European Journal of Public Health - current issue |
| The Fickle Finger of Fate |
| The Florida Times-Union - Online (jacksonville.com) |
| The Fox 105.3 |
| The Fraserburgh Herald - Online |
| The Free Library |
| The Gazette - Online |
| The Global Dispatch |
| The Globe and Mail Online |
| The Guardian |
| The Hamilton Spectator - Online |
| The Hastings Center |
| The Health Cast |
| The Health Site |
| The Herald Scotland |
| The Hindu Online |
| The Hospitalist - Online |
| The Hour - Online |
| The Huffington Post |
| The Incidental Economist |
| The Indianapolis Star - Online (indystar.com) |
| The International Information Literacies Research Network |
| The Invisible Opportunity: Hidden &hellip |
| The Irish Times - Online |
| The Japan News |
| The Japan Times Online |
| The Journal Gazette - Online |
| The Kid's Doctor: Take Charge. |
| The Lancet - Oncology |
| The Lancet (United Kingdom) |
| The Ledger - Online |
| The Liberty Beacon |
| The Med School Project |
| The National - Online (thenational.com.pg) |
| The National (www.thenational.scot) |
| The Nation's Health - Online |
| The New York Times |
| The New Yorker - Online |
| The News-Messenger Online |
| The Northern Star - Online |
| The Nursing Show |
| The Observer - Online (Uganda) |
| The Onion |
| The Ontarion |
| The Pacifican - Online |
| The paddy fields view blog |
| The Peninsula - Online |
| The Province Online |
| The Rebel Chick |
| The Register-Herald Online |
| The Reminder Online |
| The Sacramento Bee Online |
| The Sisters Club |
| The Star (Canada) |
| The Star Phoenix - Online |
| The Telegram - Online |
| The Thinking Moms' Revolution |
| The Time - Online (Beaver County) |
| The Times of Northwest Indiana - Online |
| The Times.com |
| The University Daily Kansan |
| The Vanguard - Online |
| The Vatic Project blog |
| The World 247.com |
| TheBlaze blog |
| TheCarousel.com |
| thedemonica.tumblr.com |
| TheEastEnder |
| TheHomoCulture.com |
| TheItalyNews.Net |
| TheJournal.ie |
| Theneeds |
| TheStreet |
| TheTurkeyNews.Net |
| ThinkProgress |
| Thompson Citizen - Online |
| Tiempo en Linea |
| Tiempos del Mundo |
| Times Colonist - Online |
| Times of India - Online |
| Times Recorder - Online |
| Times-Herald - Online |
| Times-Mail - Online |
| Times-Post - Online |
| Times-Standard - Online |
| Tipp FM |
| Tiroler Tageszeitung - Online |
| Tiscali Lifestyle |
| TMCnet |
| Today Online |
| Top Fash |
| TOP SANTE - ONLINE |
| Topix |
| Toronto Sun - Online |
| Totally Random Family Gal blog |
| trading-house.net |
| Tranås-Tidning - Online |
| Trelleborgs Allehanda - Online |
| Triangle Tribune - Online |
| Tribuna de Ávila |
| Tribuna de Burgos |
| Tribuna De Salamanca |
| Tribuna de Valladolid |
| Tribuna Palencia |
| Tribuna Popular |
| Tribuna Segovia |
| Tribuna Zamora |
| Tribune - Online |
| Tribune Star - Online |
| Tri-City Herald - Online |
| Trinuna Leon |
| Tristates Radio |
| Truro Daily News - Online |
| Truthout |
| [tsarcasm.tumblr.com](http://tsarcasm.tumblr.com/) |
| Ttela - Online |
| Tubantia |
| TV 2 / DANMARK (nyhederne.tv2.dk) |
| TV 2/Fyn (Denmark) |
| TV 2/Lorry - Online (Denmark) |
| TV øst (Denmark) |
| TV Wolne Media |
| TV/MIDT-VEST (Denmark) |
| TV2 oj (Denmark) |
| TV3 Ireland |
| TV4 - Online (Sweden) |
| Tweed Daily News |
| Twente FM |
| U.S. News & World Report |
| U.S. Pharmacist Online |
| UAE NewsApp.com |
| Uganda News & Information |
| Ugeskrift for Læger |
| uk.pairsonnalites.org |
| Ulitzer (PR Newswire) |
| Uloop News |
| Ulster Unionist Party.org |
| UNC Gillings School of Public Health News |
| Uncensored - Online (New Zealand) |
| Uncova.com |
| Uniradioinforma |
| United Press International Online |
| UnitedHealthcare |
| Univers smartphone |
| Universal Journal Review |
| Uno más uno - Online |
| Unorthodox-Jew blog |
| Uppsala BIO |
| Uppsala universitet |
| Upsala Nya Tidning |
| USA Partisan |
| Utah People's Post |
| Uusi Suomi (Finand) |
| UVA Today |
| UX Delaware |
| Vaccine Liberation Army |
| Vaccine Research Library |
| Vacuven blog |
| Vancity Buzz |
| Vancouver Observer |
| Vancouver Sun - Online |
| Vanguardia Liberal |
| Vanguardia MX |
| Varmatin.com |
| Vasabladet - Online |
| Västerviks-Tidningen |
| Vauva (Finland) |
| Veintitantos |
| Veol.hu |
| VERACRUZANOS.INFO |
| Vernon Bc News |
| VG Nett |
| Viborg-Folkeblad.dk |
| Victorious Stranger blog |
| Vida y Salud |
| Videnskab dk |
| VietBao.vn |
| Vindicator Online |
| ViralNewsChart |
| Virgilio |
| Virginia Public Radio |
| Visalia Times-Delta Online |
| Vitalia.cz |
| Vitals - Lifehacker |
| Viva Le magazine De Ma Mutuelle |
| Vive Sana |
| Vivreaupresent |
| VLT - Online |
| VN Express |
| Voxy.co.nz |
| vs.hu |
| Waar Maar Raar |
| WABE-FM Online |
| WAFF-TV |
| Waikato Times - Online |
| WALA-TV Online |
| WALB.com |
| WalesOnline |
| Walking by Faith blog |
| wallstreet:online |
| WAMES (Working for ME in Wales) |
| WAMU-FM Online |
| WAND TV |
| Wandtv |
| WANE-TV Online |
| WantToKnow.nl |
| WAOW-TV Online |
| Warwick Beacon Online |
| Washington Times Online |
| Wat heerst er? |
| Waterford News & Star |
| WAVE 3 News |
| WBND-TV - Online |
| WBNS-TV Online |
| WBOY |
| WBRC-TV Online |
| WBTV-TV Online |
| WCAX-TV Online |
| WCBE-FM - Online |
| WCHS-TV - Online |
| WCSH-TV - Online |
| WCSI Online |
| WDAM 7 |
| WDAZ-TV - Online |
| WDJT-TV - Online |
| WDR.de (News) |
| Webmagazín.Teraz.sk |
| WebMD.com |
| Webnoviny.sk |
| WECT TV-6 |
| Week Online |
| WEEK-TV Online |
| Weightloss blog |
| Weinformers |
| WEKU-FM - Online |
| Welsh Assembly Government |
| WESA-FM Online |
| West FM |
| Western People - Online |
| Westman Journal - Online |
| Westport Daily Voice |
| Westsound FM |
| Weyburn Review |
| Weyburn This Week - Online |
| WFAE-FM - Online |
| WFDD-FM - Online |
| WFMJ 21 - TV |
| WFSB-TV |
| WFSU - Online |
| WFXG-TV Online |
| WFXT-TV Online |
| WFYI-FM Online |
| WGBH-TV Online |
| WGEM-TV Online |
| What Doctors Don't Tell You |
| WHBL-AM Online |
| WHBQ-TV Online |
| WHDH-TV Online |
| Whistler Question - Online |
| WHO Regional Office for Africa |
| WholeHealth Chicago |
| WHO-TV Online |
| WHRO-FM Online |
| Wiadomosci ze swiata |
| Wikistrike |
| Williamson Source |
| Winnipeg Free Press - Online |
| Winnipeg Sun - Online |
| WiredGov |
| WIS News 10 |
| Wisconsin Public Radio - Online |
| WISC-TV Online |
| WISH-TV Online |
| wixx.com |
| WJAR-TV Online |
| WJCT-TV - Online |
| WK Pharma - Online |
| WKAR-FM - Online |
| WKNU.org |
| WKOW-TV - Online |
| WKU Public Radio |
| WLNE-TV Online |
| WLR FM |
| wlrh.org |
| WLS-AM |
| WLTZ 38 |
| WMCTV |
| WMCTV.com |
| WMOT-FM - Online |
| WMRA |
| WMSN-ITV |
| WNIJ-FM Online |
| WNKY-TV - Online |
| WNWN-FM Online |
| WNYC-AM Online |
| WOCHE KÄRNTEN - Online |
| wochenblatt.de |
| WOHL-TV - Online |
| Wolfsburger Allgemeine |
| Wolne Media |
| WOR-AM |
| Word Matters! |
| Works of Genius |
| World Economic Forum |
| World News (wn.com) |
| World News on The Net blog |
| World Truth.TV |
| Wowo |
| WPFO-TV Online |
| WPRI-TV Online |
| WPSD-TV - Online |
| WQOW-TV Online |
| WREX-TV - Online |
| WRKF-FM Online |
| WSBT 22 |
| WSEE |
| WSFA |
| WSVN-TV Online |
| WTHI-TV - Online |
| WTHR-TV Online |
| WTLH-TV Online |
| WTNH-TV Online |
| WTOC-TV Online |
| WTVC2-TV Online |
| WUIS-FM - Online |
| WUKY - Online |
| WUNC-FM - Online |
| WUOT |
| WUSA-TV Online |
| wutc.org |
| WUWF - Online |
| WUWM-FM - Online |
| WVA fp |
| WVIR-TV Online |
| WVPB-FM - Online |
| WVTF |
| WVVA-TV Online |
| WVXU-FM - Online |
| WWNO-FM - Online |
| www.ntvt.nl |
| WXIN-TV Online |
| WXOW-TV Online |
| WXPR-FM Online |
| Wyoming Public Media |
| WYSO-TV - Online |
| xoJane.com |
| Yahoo! (Canada) |
| Yahoo! (Colombia) |
| Yahoo! (France) |
| Yahoo! (Germany) |
| Yahoo! (India) |
| Yahoo! (Singapore) |
| Yahoo! (United Kingdom |
| Yahoo! (Unites States) |
| YLE.fi |
| Yorkton This Week |
| Yorkton This Week |
| Your News Now at 10 PM - WLIO-TV |
| Your News Wire |
| Ystads Allehanda |
| ZapLog lezersreacties |
| zdravi.e15.cz |
| Žena-in.cz |
| Zimbabwe Star |
| ZIONICA.com |
| ZIZonline.com |
| Zorgnieu |
| Zorgportaal |
| Zurbains |
